# Engineering charge density in s-block potassium single-atom nanozyme for amplified ferroptosis in glioblastoma therapy

**DOI:** 10.1016/j.mtbio.2025.101889

**Published:** 2025-05-21

**Authors:** Hongjia Zheng, Zhang Guo, Fuxiang Chen, Qi Zhong, Yongrui Hu, Chengzhong Du, Huimin Wang, Penghui Wei, Wei Huang, Dengliang Wang, Yang Zhu, Dezhi Kang

**Affiliations:** a Department of Neurosurgery, Neurosurgery Research Institute, The First Affiliated Hospital, Fujian Medical University, Fuzhou, Fujian, 350005, China; b Department of Neurosurgery, National Regional Medical Center, Binhai Campus of the First Affiliated Hospital, Fujian Medical University, Fuzhou 350212, Fujian, China; c Zhangzhou Affiliated Hospital of Fujian Medical University, Zhangzhou, Fujian, 363099, China

**Keywords:** Single-atom nanozyme, s-orbitals, Ferroptosis, Potassium, Charge density

## Abstract

Single-atom nanozyme (SAN) are emerging as a cutting-edge platform for next-generation nanozyme on account of their remarkable catalytic efficiency and well-defined electronic/geometric structures. While most SAN focus on transition metal atoms as active sites, s-block main group metals have traditionally been considered catalytically inert. Herein, we develop an s-block potassium single-atom nanozyme (K-SAN) featuring K-N_4_ active sites. The s orbital in single-atom K plays a distinct and critical role in the adsorption of intermediates, enabling single-atom K to exhibit an intermediate adsorption mode different from that of transition metals. K-SAN effectively induces ferroptosis in tumor cells by initiating a reactive oxygen species storm and depleting reductive glutathione, which leads to the accumulation of lipid peroxides and the inactivation of glutathione peroxidase 4 (GPX4). Furthermore, under 808 nm laser irradiation, the catalytic activities of K-SAN are strengthened, leading to a significant inhibition of tumor growth in vivo. Density functional theory calculations and experimental results together demonstrate the defined catalytic mechanism and impressive therapeutic effect of K-SAN, highlighting its unoccupied orbitals, which can accept electron donation-a key advantage over transition metal-based SAN. This study provides significant insights for the rational design and exploration of catalytic mechanisms in s-block SAN with potent antitumor efficacy.

## Graphical abstract

A s-block main-group potassium single-atom nanozyme (K-SAN) exhibits impressive peroxidase-like activity, converting overproduced hydrogen peroxide into hydroxyl radicals, which induces lethal lipid peroxidation (LPO). Moreover, K-SAN effectively depletes endogenous glutathione, leading to the inactivation of glutathione peroxidase 4. In addition, under 808 nm laser irradiation, the catalytic activities of K-SAN are amplified, significantly promoting LPO-mediated ferroptosis in vivo.

**Figure:**
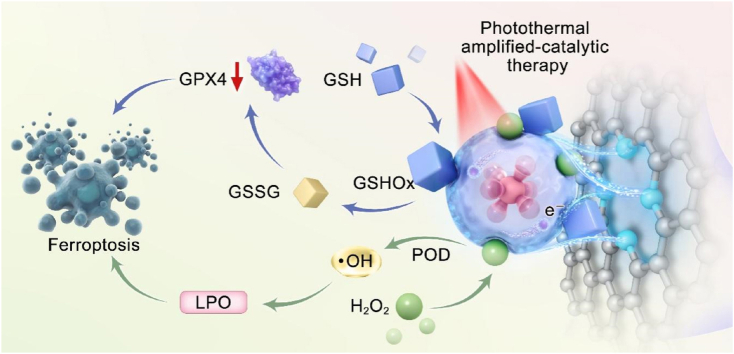

## 1 Introduction

Enzymes have been regarded as effective tools for the treatment of various diseases, with therapies leveraging their catalytic activity having the potential to transform many target molecules and restore normal physiological metabolism [[1], [2], [3]]. However, natural enzymes are arduous to prepare and highly susceptible to degradation in vivo, which significantly impeding their biomedical applications [4,5]. Fortunately, artificial enzymes can effectively address the associated challenges owing to their superior stability, low cost, and recoverability [[6], [7], [8], [9], [10]]. Nanozyme, a class of artificial nanomaterials with intrinsic enzyme-mimetic activity, have emerged as promising alternatives to natural enzymes by modulating bio-catalytic active sites at molecular level [[11], [12], [13], [14], [15]]. To date, considerable effort has been dedicated to exploring the potential of nanozyme for cancer diagnosis and therapy, advancing from in vitro studies to in vivo applications [[16], [17], [18], [19], [20]]. However, the intricate and variable crystal structures, coupled with the complex surface configurations, pose significant challenges in precisely controlling the substrate selectivity and catalytic activity of nanozyme at the molecular level [[21], [22], [23], [24]]. Furthermore, the catalytic mechanism of nanozyme remains unclear, owing to the complexity of their nanostructures, the diverse elemental composition, and the dynamic catalytic microenvironment [[25], [26], [27], [28]]. Therefore, to overcome the current challenges, there is an urgent need for rational design and precise tuning of nanozyme to achieve optimal catalytic performance, along with a defined understanding of catalytic mechanisms, to advance current biomedical sciences.

With the remarkable advancements in characterization strategies and continuous innovations in synthesis methods, the concept of “Single-atom nanozyme (SAN)” was proposed [[29], [30], [31], [32], [33], [34]]. SAN, characterized by well-defined electronic/geometric structures and optimized atom utilization efficiency, bridge the gap between artificial enzymes and nature enzymes, amplifying both intrinsic catalytic performance and substrate selectivity [[35], [36], [37], [38]]. To date, transition metals such as iron (Fe) [[39], [40], [41]], manganese (Mn) [[42], [43], [44], [45]], copper (Cu) [[46], [47], [48]], which possess sufficient d-orbital electronic states, have been widely employed in disease therapy [49]. This is due to their appropriate d-band centers enhance catalytic activity by facilitating the adsorption of intermediates during the catalytic process [50,51]. In contrast, s-block main-group metal elements, such as potassium (K) and magnesium (Mg) [[52], [53], [54]], with delocalized s/p-bands can easily broaden the adsorption state, potentially leading to overly strong or insufficient adsorption. As a result, highly active SAN based on s-block main-group metal elements have seemed unlikely for biomedical applications. However, s-block main-group metal similarly possess unoccupied orbitals capable of accommodating electron donation from oxygenated intermediates [55,56]. Recent significant advancements have been investigated the heterogeneous single-atom catalysts using alkaline earth metals as active sites offer new inspiration for the development of SAN based on s-block main-group metal elements in nanomedicine [57]. Despite these promising developments, to the best of our knowledge, there have no report on the catalytic performances of the s-block alkali metals SAN in tumor therapy.

**Scheme. 1:**
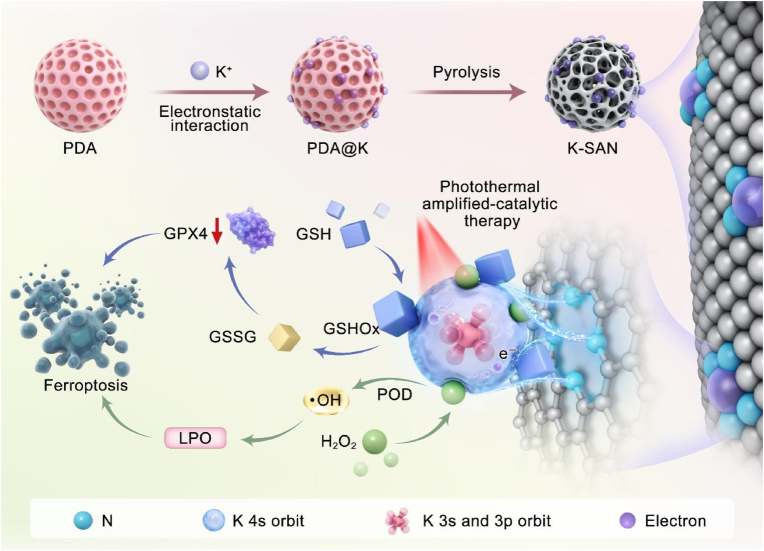
Schematic diagram of the fabrication process of K-SAN, the s orbital-induced adsorption of intermediates and tumor therapy.

Herein, we fabricate an s-block K-SAN with K-N_4_ active sites through carbonization of polydopamine (PDA) adsorbed K^+^ (K@PDA), which significant initiate highly active reactive oxygen species (ROS) storm and subsequently irreversible ferroptosis (Scheme 1). The nitrogen (N)-doped carbon (C) nanosphere is employed to anchor atomically dispersed K^*δ*+^ (0<*δ* < 1), coordinated with four N atoms. The expanded graphite layer spacing ensure substantial exposure of the active site, greatly enhance catalytic performances. K-SAN exhibits exceptional peroxidase (POD)-like activity, converting overproduced hydrogen peroxide (H_2_O_2_) into highly toxic hydroxyl radical (•OH) in the mild acidic tumor microenvironment (TME), which induces lethal lipid peroxidation (LPO). Moreover, K-SAN effectively depletes endogenous glutathione (GSH), leading to the inactivation of glutathione peroxidase 4 (GPX4) and promoting LPO-mediated ferroptosis [58]. In addition, under 808 nm laser irradiation, the catalytic activities of K-SAN are amplified, significantly suppressing tumor growth in vivo. Density functional theory (DFT) calculations and experimental results collectively reveal the defined catalytic mechanism and extraordinary therapeutic efficacy of K-SAN, which features unoccupied orbitals capable of accepting electron donation-an advantage over traditional transition metal-based SAN. This study provides valuable insights for the rational design of SAN using s-block main-group metals as catalytic centers and offers a deeper elucidating of their therapeutic mechanisms.

## 2 Result and discussion

### 2.1 Synthesis and characterization of K-SAN

**Fig. 1:**
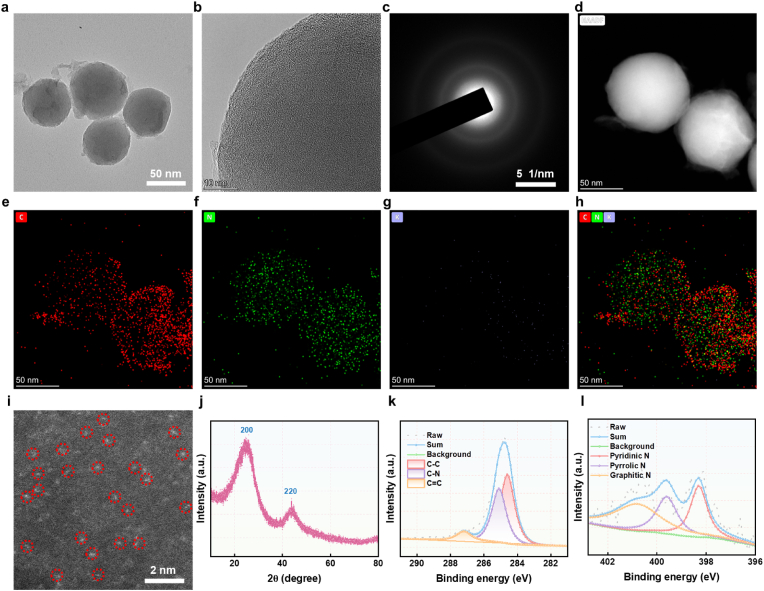
(a) TEM image of K-SAN. (b) High-resolution TEM images of K-SAN. (c) SAED pattern of the K-SAN. (d–h) EDX mapping of K-SAN. (i) Magnified HAADF-STEM image of K-SAN showing the single-atomic K as bright dots. (j) XRD pattern of K-SAN. (k) High-resolution C 1s XPS spectrum. (l) High-resolution N 1s XPS spectrum.

The synthetic route of K-SAN was shown in Scheme 1. Firstly, PDA nanospheres were obtained via oxidative polymerization using dopamine as a precursor (Fig. S1). Under alkaline conditions, the PDA carries a net negative charge, which repels anions while allowing cations to pass through. Building on this property, K^+^ ions are absorbed by electrostatic interaction, followed by pyrolysis to obtain K-SAN. Transmission electron microscopy (TEM) images showed that K-SAN exhibited a spherical morphology with a uniform nanoparticle size distribution, averaging approximately 70 nm in diameter (Fig. 1a). High-resolution TEM image revealed that the surface of K-SAN became rough and porous as a result of the high-temperature carbonization process (Fig. 1b). The corresponding selected area electron diffraction (SAED) pattern of the K-SAN displayed distinct amorphous diffraction rings, indicating the poor crystallinity (Fig. 1c). Energy-dispersive X-ray spectroscopy (EDX) mapping demonstrated a homogeneous distribution of K atoms on N-doped C frameworks (Fig. 1d–h). EDX spectrum further proved that K atoms were successfully anchored within the K-SAN (Fig. S2). The K atoms loading efficiency in K-SAN was measured as 1.1 % using inductively coupled plasma mass spectrometry (ICP-MS). Aberration-corrected atomic-resolution high-angle annular dark-field scanning transmission electron microscopy (AC HAADF-STEM) directly revealed numerous bright spots, marked with red circles, confirming the presence of single-atomic K at N vacancies (Fig. 1i). The X-ray diffraction (XRD) pattern further corroborated the crystallographic nature of the K-SAN, showing similar characteristic peaks (Fig. 1j). Notably, the absence of crystalline peaks for metallic K or K oxide nanoparticles in the XRD pattern suggested negligible K aggregation within the K-10.13039/501100008997SAN, supporting the feasibility of achieving atomically dispersed K in the K-10.13039/501100008997SAN structure. There results suggested that the successful fabrication of single-atom K species immobilized on K-SAN.

**Fig. 2:**
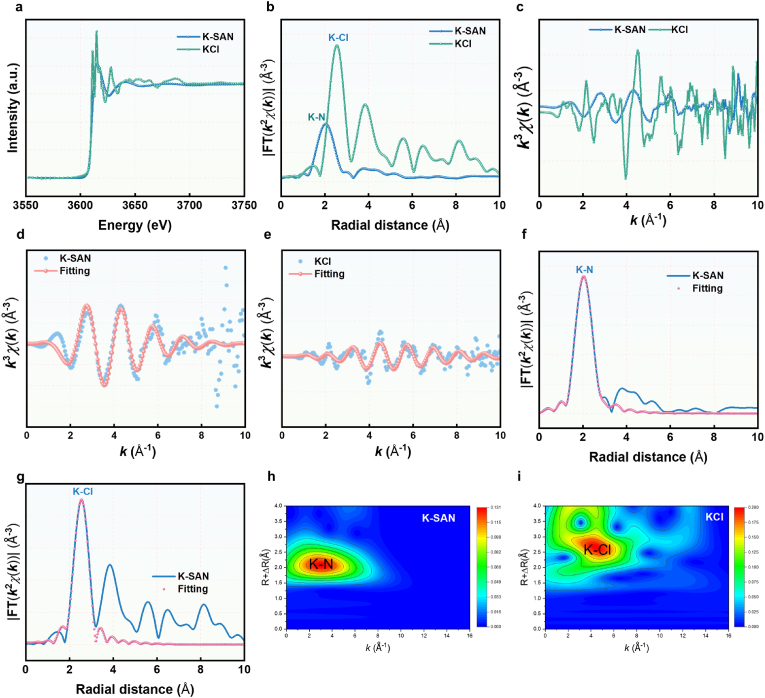
(a) K K-edge XANES spectrum of K-SAN and KCl. (b) The Fourier transform EXAFS of the K K-edge of K-SAN and KCl. (c) EXAFS curves of K-SAN and KCl at the k space. (d) EXAFS fitting curves of K-SAN and (e) KCl. (f) EXAFS fitting curve of K-SAN and (g) KCl at the R space. (H) Wavelet transformation of K K-edge EXAFS of K-SAN and (i) at the k space.

To evaluate the binding states of K and nitrogen atoms in K-SAN, X-ray photoelectron spectroscopy (XPS) analysis was conducted (Fig. 1k). As shown in Fig. 1k, the high-resolution C 1s spectrum of K-SAN predominantly showed features corresponding to sp^2^-hybridized graphitic carbon. The high-resolution N 1s spectrum demonstrated three distinct nitrogen species: graphitic N, pyrrolic N, and pyridinic N, respectively. Importantly, a high content of pyridinic N species was displayed, which offered coordination sites for immobilizing single-atomic K, potentially boosting the catalytic activity of K-SAN (Fig. 1l). In addition, the peak of K 2p at 292.6 eV (Fig. S3), indicating the existence of nature K^δ+^ (0<δ < 1) species in K-SAN. To further investigate the coordination environment of K, synchrotron radiation-based X-ray absorption near-edge structure (XANES) and extended X-ray absorption fine structure (EXAFS) analyses were performed. The K K-edge XANES spectrum exhibited an energy absorption threshold (Fig. 2a), confirming the K^δ+^ oxidation state, consistent with the XPS findings. Furthermore, the Fourier transform of the EXAFS data in the R-space revealed a clear peak at 2.0 Å corresponding to the K-N bond, confirming the atomically dispersed K active sites in K-SAN (Fig. 2b). EXAFS fitting analysis at the K K-edge provided detailed structural information, showing that the K atoms in K-SAN were coordinated with four N atoms (Fig. 2c–g, and Table S1). In addition, wavelet transform (WT) analysis was employed to further confirm the presence of atomically dispersed K species. As anticipated, K- SAN displayed a WT signal at 3.0 Å^−1^ corresponding to the K-N bond, with no WT intensity associated with K-K bonds (Fig. 2h and i). These findings conclusively revealed the successful constructions of K-SAN with atomically dispersed K-N_4_ sites.

**Fig. 3:**
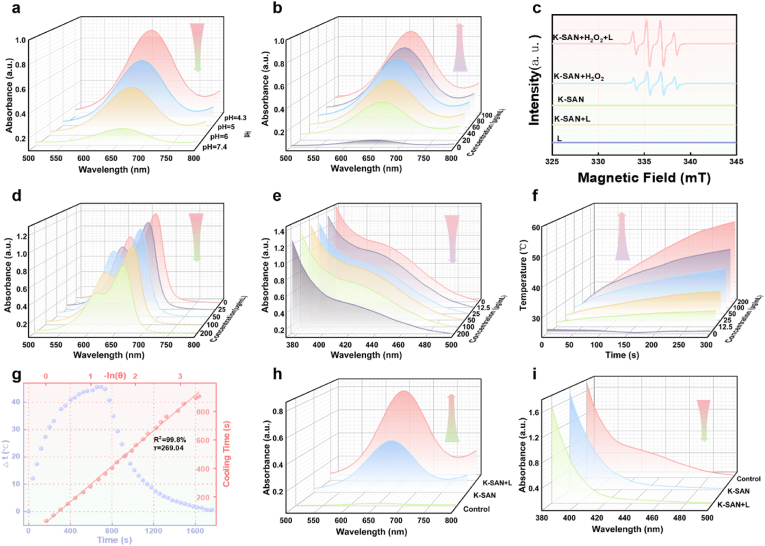
(a) UV–vis spectra of TMB incubated with K-SAN and H_2_O_2_ at various pH. (b) UV–vis spectra of TMB incubated with H_2_O_2_ in the presence of different concentrations of K-SAN. (c) The ESR curves of •OH captured using a DMPO. (d) The UV–vis spectra of MB treated with K-SAN plus H_2_O_2_ under acidic pH. (e) UV–vis spectra of DTNB incubated with K-SAN in the presence of GSH. (f) The photothermal effect of various K-SAN concentrations under laser irradiation. (g) Photothermal conversion efficiency calculation of K-SAN. (h) Fenton-like reactivity and (i) GSH depletion abilities of K-SAN under laser irradiation.

After characterizing the coordination number of K atoms in K-SAN, we systematically assessed its enzyme-like activities, including peroxidase POD- and glutathione oxidase (GSHOx)-like activities. The 3,3′,5,5′-tetramethylbenzidine (TMB) kit was used to measure the POD-mimicking activity of K-SAN. When K-SAN was combined with H_2_O_2_ a rapid color change of TMB occurred at acidic pH (from 4.5 to 6.5), but not at neutral pH (7.4), indicating the pH-dependent catalytic performance of K-SAN and the efficient conversion of H_2_O_2_ into •OH by K-SAN under mild acidic conditions (Fig. 3a and S4). Moreover, the catalytic activity of K-SAN was found to be concentration-dependent (Fig. 3b and S5). To further investigate the generation of intermediate •OH, we employed dimethyl-1-pyrroline N-oxide (DMPO) as a spin trap and conducted electron spin resonance (ESR) analysis. The ESR spectrum revealed a characteristic quartet signal (1:2:2:1) of the DMPO/•OH adduct when K-SAN was treated with H_2_O_2_ at acidic pH (Fig. 3c). Furthermore, methylene blue (MB) was carried out to evaluate the efficiency of •OH production. As depicted in Fig. 3d and S6, the absorbance significantly decreased with increasing concentrations of K-SAN when MB was incubated with K-SAN and H_2_O_2_ under acidic conditions, further demonstrating the excellent capacity of K-SAN to generate substantial amounts of •OH. As shown in Figs. S7 and S8, K-SAN demonstrated significantly higher enzymatic activity compared with other nanozymes. In addition, the GSHOx-like activity of K-SAN was measured using 5,5′-dithiobis-(2-nitrobenzoic acid) (DTNB) probe. As shown in Fig. 3e and S9, the absorbances at 412 nm decrease greatly in a concentration-dependent manner, indicating the impressive GSH depletion ability of K-SAN. The POD-like enzyme activities and GSHOx-like enzyme activities of K-SAN were both quantified. Based on the experimental data (Figs. S10 and S11), the Vmax for the POD-like activity of K-SAN was determined to be 5.87 × 10^−8^ M s^−1^, Km was calculated as 0.095 mM, and Kcat was found to be 5.7 × 10^4^ s^−1^. the Vmax for the GSHOx-like activity of K-SAN was determined to be 5.68 × 10^−8^ M s^−1^, Km was calculated as 0.519 mM, and Kcat was found to be 5.51 × 10^4^ s^−1^. In addition, control nanomaterials without potassium doping were included in this study, and their POD-like enzyme activity and GSHOx-like enzyme activity were measured. The results are presented in Figs. S12 and S13, indicating that the nanomaterials without potassium doping exhibited negligible enzyme-like activity. These results demonstrate that potassium plays an essential role in the catalytic process of nanozymes. Beyond its excellent catalytic performance, the amorphous C doped N framework in K-SAN endowed it photothermal properties. The UV–Vis absorption data of K-SAN under various wavelength illuminations, presented in Fig. S14, further substantiate the photothermal therapy capability of K-SAN. Infrared thermal imaging demonstrated that the temperature of the PBS solution showed no significant increase under 808 nm laser irradiation. In contrast, the temperature of the K-SAN solution rapidly rose to 60 °C within 5 min (Fig. 3f and S15). Repeated illumination experiments demonstrated that K-SAN exhibited superior photothermal stability after three cycles (Fig. S16). The photothermal conversion efficiency of K-SAN was calculated to be η = 27.8 % (Fig. 3g). Moreover, the catalytic performance of K-SAN was further enhanced by superior photothermal effect. As shown in Fig. 3h and i, and S17, S18), K-SAN significantly amplified the oxidation of TMB and the consumption of GSH upon 808 nm laser irradiation, demonstrating its substantial potential for tumor treatment. Collectively, these results suggested that K-SAN possesses superior catalytic properties and held great promise for initiating and amplifying ROS storms. Long-term storage stability experiments in 14 days were also supplemented as shown in Fig. S19 as below. The catalytic activities of K-SAN demonstrated excellent long-term storage stability. Dynamic Light Scattering (DLS) was employed to assess the long-term stability of K-SAN in biological systems. As shown in Fig. S20, the DLS results demonstrate that K-SAN maintains a stable particle size in the biological system over an extended period.

**Fig. 4:**
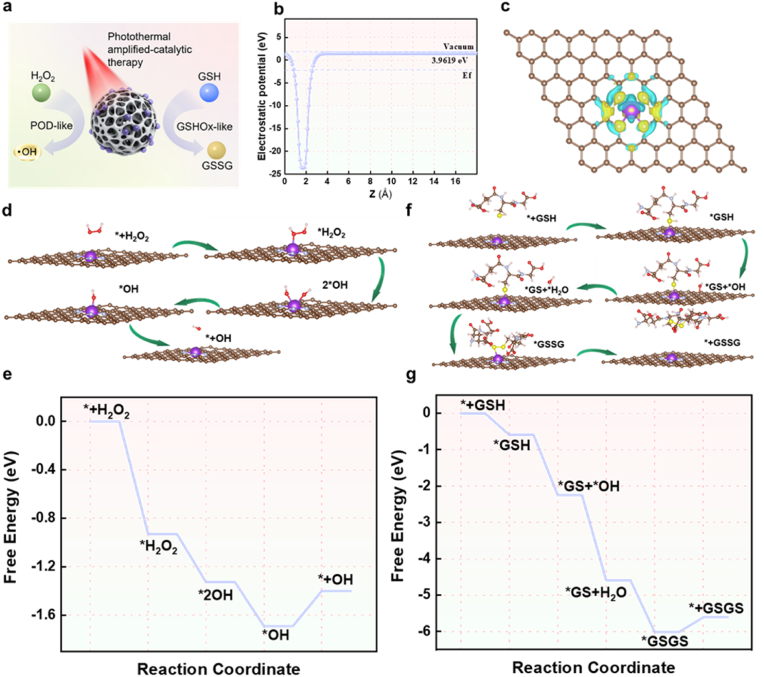
(a) Schematic diagram of POD- and GSHOx-like activities of K-SAN. (b) The work functions spectra of K-SAN. (c) The local charge density difference plot of the K-SAN. Yellow: charge accumulation; Cyan: charge depletion. (d) DFT optimized structures of the •OH intermediates on K-SAN surface. (e) Gibbs free-energy diagrams for POD-like activity on the modeled surface of K-SAN. (f) DFT optimized structures of the GSSG intermediates on K-SAN surface. (g) Gibbs free-energy diagrams for GSHOx-like activity on the modeled surface of K-SAN. (For interpretation of the references to color in this figure legend, the reader is referred to the Web version of this article.)

To further elucidate the mechanism behind the POD- and GSHOx-like activities of K-SAN induced by s orbitals in K, we performed DFT calculations (Fig. 4a). The nanostructures were modeled and simulated using a defect crystal approximation representation (Fig. S21). We calculated the work functions of K-SAN along with the charge density difference to investigate the interface characteristics and charge distribution between C-N and single-atomic K. The work functions of K-SAN were calculated to be 3.9619 eV (Fig. 4b), leading to an equilibrium at the Fermi level. The charge density difference pattern of K-SAN further demonstrated the charge distribution between C-N and single-atomic K (Fig. 4c), indicating a strong interaction at the interface, which in consistent with XPS results. The significant charge transfer from C-N to the K 4s orbitals revealed that the built-in electric field boosted the effective adsorption of intermediates. For the POD-mimicking activity, the geometrically optimized H_2_O_2_ was easily absorb onto the single-atomic K of K-SAN (Fig. 4d and e). The activated H_2_O_2_ was dissociated homogeneously to generate two reactive ∗OH species on the K 4s orbitals. The absorbed OH∗ then reacted with protonated hydrogen atom to form H_2_O, which was adsorbed on the K-N_4_ active sites in a mildly acidic environment. Afterward, the absorbed ∗OH reacted with protonated H atom to form H_2_O on the K-N_4_ active sites. Finally, the K-SAN returned to its initial state following the desorption of H_2_O, leading to an obvious decline of the Gibbs free energy. For GSHOx-mimicking activity, the optimized GSH molecules spontaneously absorbed onto the single-atomic K (Fig. 4f and g). Mechanically, the absorbed GSH∗ and OH∗ reacted to form GS∗ on K 4s orbitals, accompanied by the regeneration of H_2_O. The generated GS∗ can then couple with another GSH molecule to form GSSG. To our best knowledge, this work first presented reasonable catalytic mechanisms for POD- and GSHOx-mimicking activities of an s-block K-SAN. Thus, the DFT results provide further insights into the efficient POD- and GSHOx-like activities of K-SAN.

### 2.2 Therapeutic efficacy of K-SAN in tumor cell

**Fig. 5:**
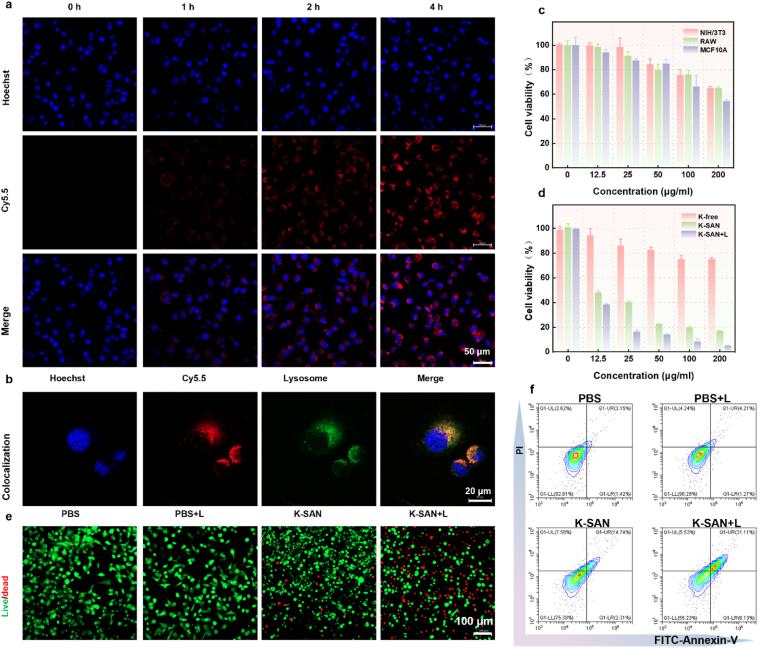
(a) The CLSM images of GL261 cells treated with Cy5.5-labeled K-SAN. (b) The CLSM images of GL261 cells colocalization. (c) The cell viability of non-cancerous cells after 24 h treatment under different K-SAN concentrations. (d) Tumor cell viability after 24 h of incubation with varying concentrations of K-SAN. (e) The fluorescence images of live/dead staining for GL261 cells incubated with varying formulations. (f) Flow cytometry measurements of GL261 cells after incubation with different formulations. ∗∗∗∗P < 0.0001.

Building on its excellent photothermal-amplified catalytic activities, the antitumor effect of K-SAN was evaluated in vitro using Glioma 261 (GL261) cells. To assess cellular uptake, cyanine 5.5 (Cy5.5)-labeled K-SAN was employed, and confocal laser scanning microscopy (CLSM) showed a time-dependent increase in red fluorescence within GL261 cells (Fig. 5a). Additionally, results from the colocalization experiment indicated that K-SAN localized predominantly in lysosomes and cytoplasm (Fig. 5b), As shown in Fig. S22, the H_2_O_2_ level in GL261 cells was significantly higher than that in normal cells. which aligned with the acidic conditions necessary to enhance K-SAN-medicated Fenton reaction. The cell counting kit-8 (CCK-8) was used to quantitatively assess the in vitro antitumor effect of K-SAN on nonneoplastic cells. As can be seen in Fig. 5c, K-SAN had negligible impact on RAW, 3T3, and MCF7-10A cells, ascribed to significantly low endogenous H_2_O_2_ levels in noncancerous cells. The CCK-8 experimental results showed a significant decrease in the survival rate of GL261 cells with increasing concentrations of K-SAN (Fig. 5d). Importantly, the survival rate of tumor cells following treatment with K-SAN combined with laser irradiation was markedly lower than that of K-SAN group. However, tumor cell death was negligible in the potassium-free material. This strongly corroborates the photothermal therapeutic efficacy of K-SAN while demonstrating that potassium plays a critical catalytic role in K-SAN. Additionally, the propidium iodide (PI)/calcein-AM co-staining assay was employed to visually assess the antitumor effect of K-SAN. As expected, K-SAN-treated cells exhibited a significantly higher rate of apoptosis compared to the control group, with laser irradiation further augmenting the cytotoxicity of K-SAN on tumor cells (Fig. 5e and S23). Furthermore, flow cytometry analysis was conducted to assess the anti-proliferative effect of K-SAN on GL261 cells. The results indicated that K-SAN-treated cells exhibited a significantly higher percentage of cell death compared to the control group, and the tumor cell death rate was further amplified by the combination of K-SAN treatment and laser irradiation (Fig. 5f). These findings corroborated that K-SAN effectively inhibited tumor proliferation, leveraging its photothermal-amplified catalytic activities.

**Fig. 6:**
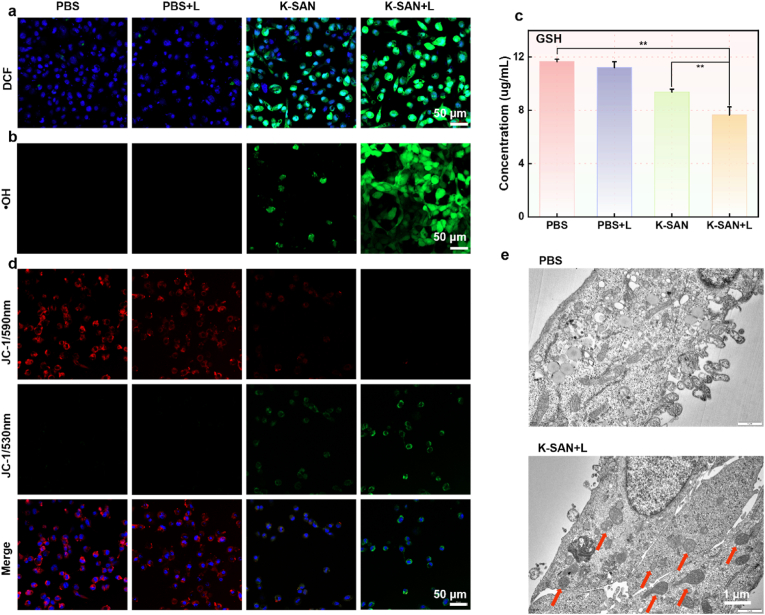
(a) DCF fluorescence images of GL261 cells following varying treatments. (b) The fluorescence images of •OH probe O26-stained GL261 cells following varying treatments. (C) The GSH levels in GL261 cells following varying treatments. (d)The CLSM images of MMP in GL261 cells following varying treatments. (e) Bio-TEM of GL261 cells treated with K-SAN. ∗∗∗∗P < 0.0001.

In light of the POD-like activity of K-SAN and its photothermal properties, a fluorescent probe (2′,7′-dichlorofluorescein diacetate, DCFH-DA) was employed to assess the levels of ROS levels in GL261 cells. The experimental results demonstrated that the K-SAN group significantly elevated levels of ROS, and 808 nm laser irradiation further augmented ROS levels in GL261 cells (Fig. 6a and S24, 25). The elevation of intracellular ROS can be ascribed to the generation of •OH, and the •OH-specific fluorescence probe O26 was employed to confirm this. CLSM images further corroborated that K-SAN was highly effective in promoting •OH production, with laser irradiation significantly boosting this effect (Fig. 6b and S26, 27). It is important to emphasize that GSH plays a crucial role in mitigating the ROS levels. Fortunately, K-SAN demonstrated a substantial capacity to deplete GSH and induce oxidative stress, leading to mitochondrial damage (Fig. 6c and S28). To evaluate mitochondrial membrane potential (MMP) polarization, a mitochondria probe, 5,5′,6,6′-tetrachloro-1,1′,3,3′-tetraethyl-imida-carbocyanine iodide (JC-1) was used, which monitors its fluorescence shift from red to green. As illustrated in the CLSM image (Fig. 6d), the intensity of green fluorescence in the K-SAN group was significantly higher than that in the control group. Laser irradiation further enhanced the green fluorescence intensity in the K-SAN group, while the red fluorescence intensity exhibited an inverse pattern. Furthermore, biological TEM (Bio-TEM) of tumor cells treated with K-SAN revealed mitochondrial damage, a hallmark of ferroptosis (Fig. 6e). These findings confirmed the polarization of MMP in GL261 cells, suggesting that K-SAN treatment may induce tumor cell ferroptosis.

**Fig. 7:**
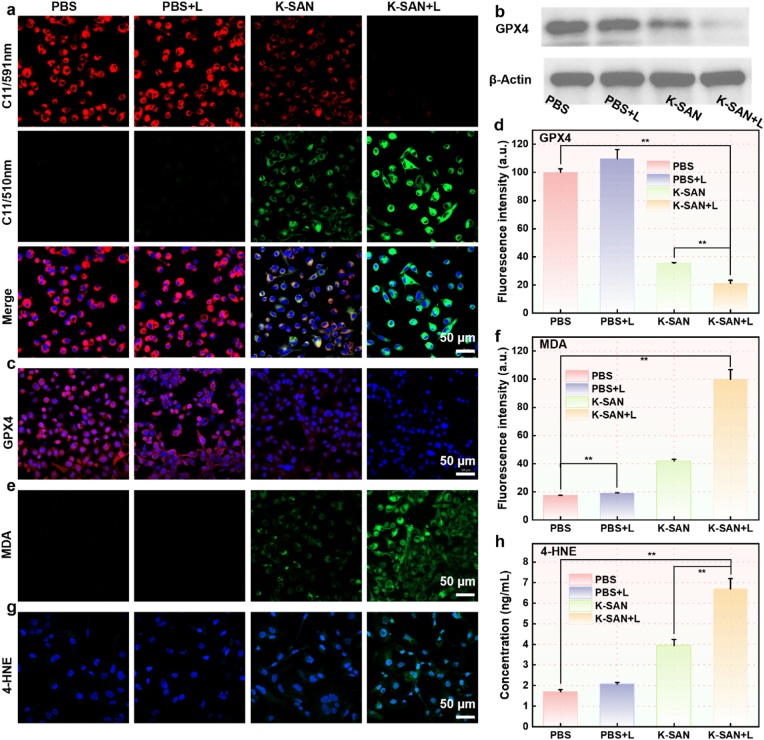
(a) The confocal images of fluorescent indicator C11-BODIPY^581/589^-stained GL261 cells following varying treatments. (b) GPX4 expression in GL261 cells incubated with varying formulations by WB. (c) The immunofluorescence images and (d) corresponding quantification of GPX4 expression in GL261 cells following varying treatments. (e) The confocal images and (f) the corresponding quantification of MDA levels in GL261 cells following varying treatments. (g) The confocal images of 4-HNE levels in GL261 cells following varying treatments. (h) 4-HNE levels in GL261 cells following varying treatments. ∗∗∗∗P < 0.0001.

Building on those experimental results presented above, we further validated key indicators of ferroptosis. As a potent oxidant, highly reactive •OH can oxidize polyunsaturated fatty acids and lead to the accumulation of lipid peroxides. The lethal accumulation of LPO and the inactivation of GPX4 are characteristic features of ferroptosis. A fluorescent indicator, C11-BODIPY^581/589^, was used to monitor LPO in cell membranes. K-SAN treatment resulted in a significant enhancement of green fluorescence, accompanied by a gradual reduction in red fluorescence, indicating that induction of LPO accumulation, which was further intensified by laser irradiation (Fig. 7a). Simultaneously, the expression levels of GPX4 in GL261 cells were assessed. As illustrated in Fig. 7b, western blot (WB) analysis demonstrated that K-SAN treatment induced a downregulation of GPX4 expression, a finding further corroborated by immunofluorescence results (Fig. 7c and d and S29). Furthermore, we assessed additional ferroptosis indicators, including malondialdehyde (MDA) and 4-hydroxynonenal (4-HNE), The combination of K-SAN and laser irradiation significantly elevated the levels of MDA and HNE in GL261 cells (Fig. 7e–h, and S30-33). These results indicated that K-SAN induced ferroptosis in tumor cells via the accumulation of LPO and the inactivation of GPX4.

### 2.3 In vivo antitumor efficacy of K-SAN

**Fig. 8:**
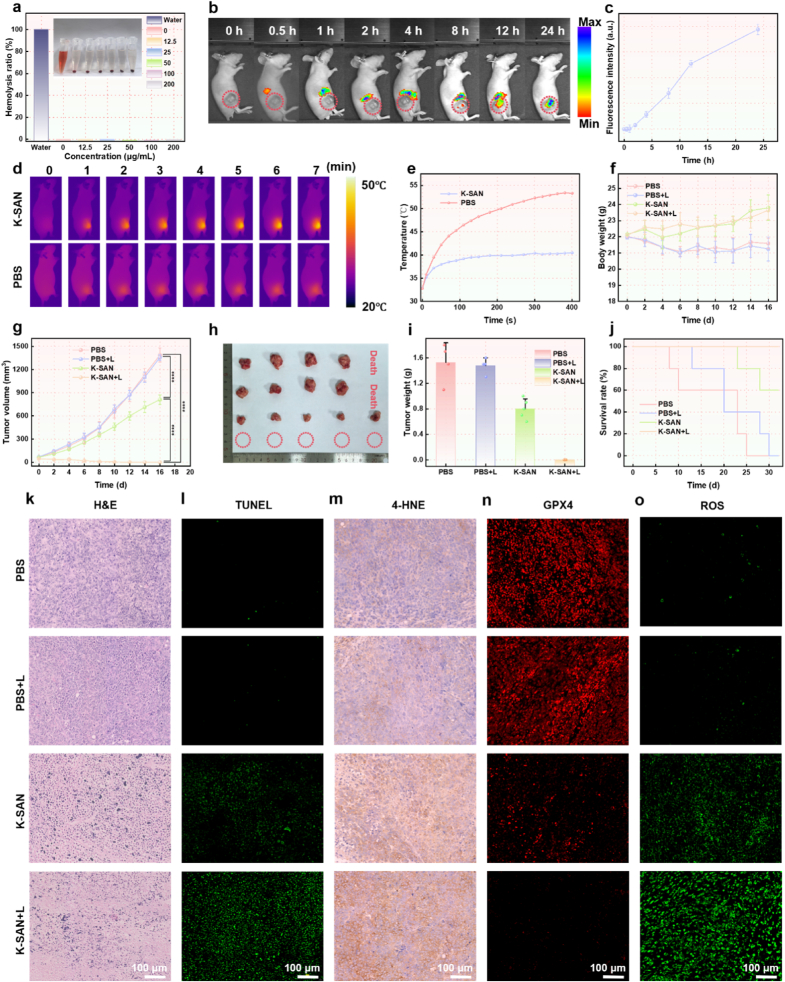
(a) The percentages of hemolysis in response to different concentrations of K-SAN (Inset: photograph of centrifuge tubes containing the supernatant from erythrocytes exposed to DI water or different concentrations of K-SAN in PBS). (b) The fluorescence images and (c) corresponding quantification of GL261 tumor-bearing mice at various time points following the injection of Cy5.5-labeled K-SAN. (d) The thermal images and (e) corresponding tumor temperature variations upon laser irradiation. (f) The body weight curves of mice following varying treatments. (g) The tumor growth curves of GL261 tumor-bearing mice following varying treatments. (h) The images and (i) tumor weights were collected from mice at the conclusion of the treatment. (j) The Kaplan-Meier survival profiles of mice following varying treatments. (k) H&E, (l) TUNEL staining of tumor slides were harvested from varying groups. (m) The immunohistochemical analysis of 4-HNE levels in tumors obtained from varying groups. (n) The immunofluorescence staining of GPX4 expressions and (o) ROS levels. ∗∗∗∗P < 0.0001.

Animal experiments were performed according to the protocol (IACUC FJMU2022-0608) approved by The Ethical Committee of Fujian Medical University. The biosafety of K-SAN was assessed prior to evaluating its therapeutic efficacy in mice. Hemolysis assays conducted across various concentrations of K-SAN (below 1000 μg/ml) showed negligible hemolysis, indicating that K-SAN exhibits favorable biocompatibility (Fig. 8a). All animal experimental protocols were approved in advance by the Ethics Committee of Fujian Medical University. Furthermore, the biosafety of K-SAN catalyst was assessed using routine blood examinations. No evident variety in blood indices could be observed after treating the mice with K-SAN (Fig. S34).Hematoxylin and eosin (H&E) staining was employed to examine the normal cellular morphology of each tissue in the mice, indicating no significant histological damage (Fig. S35). The IVIS imaging system was utilized to assess K-SAN accumulation in mice. Cy5.5-labeled K-SAN was intravenously injected, and fluorescence imaging (FLI) was presented in Fig. 8b and c. The fluorescence intensity in the tumor region continuously amplified and maintained a high intensity for up to 24 h post-injection, suggesting substantial tumor accumulation of K-SAN, likely owing to the enhanced permeability and retention (EPR) effect (Fig. S36). Subsequently, leveraging the excellent near-infrared photothermal property of K-SAN, 808 nm laser irradiation (1 W/cm^2^) was applied to the tumor site 24 h after intravenous administration, followed by near-infrared thermal imaging. Experimental results demonstrated a rapid temperature increase at the tumor region in comparison to PBS group, indicating that K-SAN exhibited a strong photothermal effect (Fig. 8d and e).

Given its photothermal properties and remarkable catalytic activities, the in vivo antitumor efficacy of K-SAN was assessed. GL261-bearing nude mice were allocated into four groups: control, laser, K-SAN, and K-SAN plus laser. As shown by body weight measurements (Fig. 8f), neither the K-SAN group nor the K-SAN plus laser group exhibited significant weight changes, further confirming the excellent biocompatibility of K-SAN. The progression of tumor growth was monitored, and the results are presented in Fig. 8g and S37. Basedon the capacity of K-SAN to induce ferroptosis through the inactivation of GPX4 and the promotion of LPO accumulation, led to partial inhibition of tumor growth following K-SAN treatment. Importantly, the K-SAN plus laser group exhibited a more pronounced tumor inhibitory effect, which can be attributed to its unique photothermal-enhanced catalytic activities (Fig. 8h and i). Kaplan-Meier survival curves of mice treated with different formulations indicated that K-SAN treatment significantly improved the survival rate and exhibited a considerable therapeutic effect on tumors (Fig. 8j). The anti-tumor effect of K-SAN was further validated through H&E and Terminal deoxynucleotidyl transferase dUTP nick end labeling (TUNEL) staining of tumor sections. H&E staining revealed that tumor tissues from mice treated with K-SAN plus laser exhibited the most severe damage, underscoring the remarkable therapeutic effect of K-SAN (Fig. 8k). Additionally, TUNEL staining showed the highest level of cell death in the combination treatment group (Fig. 8l and S38). Fur thermore, the immunohistochemical staining results showed the highest expression levels of the 4-HNE following treatment with the K-SAN combined with laser irradiation, suggesting that K-SAN promotes LPO accumulation and subsequent ferroptosis (Fig. 8m). Immunofluorescence staining revealed a significant reduction in GPX4 expression in tumor cells within the K-SAN combined with laser irradiation group (Fig. 8n and S39). Immunofluorescence staining revealed a significant elevation in ROS levels in tumor cells in the group treated with K-SAN in combination with laser irradiation (Fig. 8o and S40). These f indings the role of K-SAN in generating ·OH, triggering a ROS storm, depleting GSH, and promoting LPO accumulation, ultimately resulting in ferroptosis of tumor.

## 3 Conclusion

In conclusion, this work presented the first development of an s-block main-group metal K-SAN featuring a K-N_4_ configuration. DFT calculations s orbital plays a crucial role in intermediate adsorption, enabling K to exhibit a unique mode of intermediate interaction. The simulations further indicated that the K-N_4_ active site is key to for the enhanced catalytic activity of K-SAN, facilitating •OH production by lowering the energy barrier at the rate-determining step in the H_2_O_2_ heterogeneous cleavage pathway. Meanwhile, K-SAN was found to deplete elevated levels of GSH. Moreover, the superior photothermal properties of K-SAN are attributed to its amorphous carbon-doped nitrogen structure, which significantly amplified the catalytic performance. Both in vitro and in vivo experimental results provided a comprehensive investigation into the mechanism underlying tumor cell death induced by K-SAN, revealing its ability to initiate ROS storms and deplete GSH, thereby inducing irreversible LPO-mediated ferroptosis. This study offered significant insights for the rational design of SAN with s-block main-group metals as catalytic centers and for deepens our understanding of their therapeutic mechanisms.

## CRediT authorship contribution statement

**Hongjia Zheng:** Conceptualization. **Zhang Guo:** Data curation. **Fuxiang Chen:** Formal analysis. **Qi Zhong:** Methodology. **Yongrui Hu:** Resources. **Chengzhong Du:** Software. **Huimin Wang:** Resources. **Penghui Wei:** Formal analysis. **Wei Huang:** Investigation. **Dengliang Wang:** Software. **Yang Zhu:** Writing – review & editing, Writing – original draft, Supervision. **Dezhi Kang:** Writing – review & editing, Writing – original draft, Supervision.

## Ethics approval and consent to participate

Animal experiments were performed according to the protocol approved by The Ethical Committee of Fujian Medical University (IACUC FJMU2022-0608).

## Declaration of competing interest

The authors declared that they have no known competing financial interests or personal relationships that could have appeared to influence the work reported in this paper.

## Data Availability

Data will be made available on request.
