# Supplementary material for: Engineering charge density in s-block potassium single-atom nanozyme for amplified ferroptosis in glioblastoma therapy
Source: Mater Today Bio. 2025 May 21;32:101889. doi: 10.1016/j.mtbio.2025.101889 (PMC12159517; doi:10.1016/j.mtbio.2025.101889)
Supplement: Multimedia component 1 [file mmc1.docx]

Supporting Information

**Engineering charge density in s-Block potassium single-atom nanozyme for amplified ferroptosis in glioblastoma therapy.**

Hongjia Zheng^+^, Zhang Guo^+^, Fuxiang Chen, Qi Zhong, Yongrui Hu, Chengzhong Du, Huimin Wang, Penghui Wei, Wei Huang, Dengliang Wang*, Yang Zhu* and Dezhi Kang*

**Chemicals**

Potassium chloride (KCl), 2,2’-azino-bis(3-ethylbenzothiazoline-6-sulfonic acid) diammonium salt (ABTS), hydrofluoric acid (HF), paraformaldehyde (PFA), Tween 20, Triton X-100, trimethylol aminomethane (Tris), dopamine, hydrogen peroxide (H_2_O_2_), acetic acid (HAc), sodium acetate (NaAc), ammonium hydroxide, and ethanol were purchased from Sinopharm Chemical Reagents (Shanghai, China). 3,3',5,5'-tetramethylbenzidine (TMB), and C11-BODIPY^581/591^ were provided by Sigma-Aldrich (St. Louis, USA). Hoechst 33342, 2′,7′-dichlorofluorescin diacetate (DCFH-DA), cell count kit-8 (CCK-8), annexin V-FITC/PI apoptosis detection kit, thiobarbituric acid (TBA), AM/PI, and 1,1',3,3'-tetraethyl-5,5',6,6'-tetrachloroimidacarbocyanine iodide (JC-1) were bought from Beyotime (Shanghai, China). Dulbecco's modified eagle medium (DMEM) was purchased from Hyclone (Logan, USA). Hydroxyl radical assay kit (O26 probe) was provided by Beijing BioRab Technology Co. Ltd. (Beijing, China). 5,5-dimethyl-1-pyrroline N-oxide (DMPO) was bought from Dojindo (Dojindo, Japan). Cyanine 5.5 monosuccinimidyl ester (Cy5.5-NHS), ELISA, and annexin V-FITC/PI apoptosis detection kit were purchased from Beijing Solarbio Science & Technology Co., Ltd. (Beijing, China). Live & Dead Bacterial Staining Kit (Cat#40274ES60) and GMyc-PCR Mycoplasma Test Kit (Cat#40601) was purchased from Yeasen Biotechnology (Shanghai) Co., Ltd.. 20 mm glass-bottom dishes, and centrifuge tubes were obtained from NEST Biotechnology Co. Ltd. (Wuxi, China). Deionized (DI) water was obtained from a Milli-Q water purification system.

**Instruments**

Powder X-ray diffraction (XRD) patterns were recorded on a Rigaku Miniflex-600 diffractometer. Transmission electron microscope (TEM) images were taken by Hitachi-7700. High-angle annular dark field scanning transmission electron microscopy (HAADF-STEM) images were recorded by JEM-ARM200F (JEOL) TEM/STEM with a spherical aberration corrector. The energy-dispersive X-ray spectroscopy (EDS) mapping was performed by JEM-2100F. X-ray photoelectron spectroscopy (XPS) spectra were collected on scanning X-ray microprobe (PHI 5000 Verasa, ULAC-PHI). Scanning electron microscopy (SEM) images were taken by Nova NanoSEM 230. Fluorescence imaging was performed by confocal microscopy (Nikon C2). The absorption spectra were measured by a ultraviolet-visible (UV-vis) UH4150 spectrophotometer (Hitachi). Metal content was measured by using inductively coupled plasma mass spectrometer (ICP-MS, PlasmaQuad 3, Thermo Elemental). Hydrodynamic diameters and zeta potentials were determined by a Zetasizer nano ZS instrument (Malvern). Cancer cell apoptosis was monitored by using a flow cytometer (CytoFLEX, Beckman).

**Synthesis of K-SAN**

In a typical procedure, 3.5 g of tris(hydroxymethyl)methyl aminomethane was dissolved in a mixture of 360 mL of distilled water (DI water) and 240 mL of anhydrous ethanol, followed by the rapid addition of dopamine hydrochloride (600 mg) and vigorous stirring at room temperature for 20 h. The resulting precipitate was collected by centrifugation and dispersed in 30 mL of deionized water, followed by the addition of 0.5 g of KCl and stirring overnight. The precipitate was washed twice with deionized water and ethanol, dried at 60 °C, ad pyrolyzed at 800 °C with a heating rate of 5 °C min^-1^ for 2 h in an N_2_ atmosphere.

**Hydroxyl radicals (•OH) generation by K-SAN mediated catalytic reaction**

The catalytic activity of K-SAN was measured by using TMB as a probe which can be converted into oxidized TMB with blue color by •OH. Briefly, Different concentrations of K-SAN and H_2_O_2_ (100 μM) were successively added into the PBS solutions with TMB (40 ug/mL) , and the mixtures were shaken at 37 °C for 10 min. After centrifugation, the absorption spectra of supernatant were measured.

K-SAN and H_2_O_2_ (100 μM) were successively added into the TMB (40 ug/mL) solutions with different pH values, and the mixtures were shaken at 37 °C for 10 min. After centrifugation, the absorption spectra of supernatant were measured.

Electron spin resonance (ESR) analysis was carried out using DMPO as the spin trapper. To confirm the •OH generation. 10 mM NaAc-HAc buffer solution (pH 5) containing 5 mM H_2_O_2_, K-SAN (50 μg/mL), and 100 mM DMPO was ultrasonicated for 1 min. Then, the mixture was transferred to a quartz tube for ESR measurement.

K-SAN and H_2_O_2_ (10 mM) were mixed with MB (20 ug/mL) solutions at acidic pH, and the mixtures were shaken at 37 °C for 10 min. After ultrafiltration, the •OH-induced MB degradation was measured by the absorbance change at 500-800 nm.

**GSH consumed by K-SAN mediated catalytic reaction**

K-SAN and H_2_O_2_ (100 uM) were mixed with GSH (10 mM) solutions at acidic pH, and the mixtures were shaken at 37 °C for 30 min. Finally, the DTNB (0.5 mg/ml) solution was added. After ultrafiltration, the GSH depletion was measured by the absorbance change at 380-500 nm.

**Calculation of the photothermal conversion efficiency (PCE)**

The PCE of K-SAN was measured following Roper’s method. 1.0 mL K-SAN (100 μg/mL) was added in a glass vial and irradiated by 808 nm laser (1.0 W cm^-2^) for 600 s, then turn off the laser until the solution cooled to room temperature. The temperature of K-SAN was monitored by a thermocouple microprobe submerged in the solution. The temperature curves were recorded to calculation of the photothermal conversion efficiency (PCE).

Different concentrations of K-SAN was added in a glass vial and irradiated by 808 nm laser (1.0 W cm^-2^) for 600 s, The temperature of K-SAN was monitored by a thermocouple microprobe submerged in the solution. The temperature curves were recorded. The experiment was repeated three cycles to investigate the photothermal stability of the material.

Two parts of the same concentration of K-SAN and one part of PBS were successively added into the PBS solutions with TMB (40 ug/mL) and H_2_O_2_ (100 μM), One bottle of K-SAN and one bottle PBS irradiated by 808 nm laser (1.0 W cm^-2^) for 600 s and other mixtures were shaken at 37 °C for 10 min. After centrifugation, the absorption spectra of supernatant were measured.

Two parts of the same concentration of K-SAN (200 μM) and one part of PBS were successively added into the PBS solutions with GSH (10 mM) and H_2_O_2_ (100 μM), One bottle of K-SAN and one bottle PBS irradiated by 808 nm laser (1.0 W cm^-2^) for 600 s and other mixtures were shaken at 37 °C for 10 min. Finally, the DTNB (0.5 mg/ml) solution was added. After centrifugation, the absorption spectra of supernatant were measured.

**Cellular uptake of Cy5.5-labeled K-SAN**

GL261 cells were seeded in confocal dishes for 12 h. After 4 h of incubation with Cy5.5-labeled K-SAN, the cells were co-stained with 20 μM Hoechst and 10 μM lysome-Tracker for 20 min. The fluorescence imaging of tumor cells was imaged by CLSM.

**Cytotoxicity assessments**

Live/dead cell staining assay was monitored by CLSM to observe the toxicity directly. The GL261 cells were seeded in confocal dishes and incubated for 12 h. After 24 h of exposure to K-SAN without/with laser irradiation (1 W/cm^2^, 808 nm), the cells were co-stained with calcein-AM and PI for 20 min. The fluorescence imaging of cells was observed directly by confocal microscopy.

Cell-viability was determined by the CCK-8 assay. For CCK-8 assay, GL261 cells were planted for 24 h. Then, the cells were incubated with various concentrations of K-SAN without/with laser irradiation. After treatment for 24 h, the medium was replaced with fresh medium containing 10 μL CCK-8 and quantified by the absorbance at 450 nm using a microplate reader.

For analysis of cell death, Annexin V-FITC and PI kit was conducted. GL261 cells were seeded and incubated 12 h. Subsequently, the cells were exposed to K-SAN without/with laser irradiation. After co-staining with Annexin V-FITC and PI according to the manufacturer’s protocols. The quantitative cell death was analyzed by flow cytometry.

***In vitro* reactive oxygen species (ROS) generation**

GL261 cells were seeded in confocal dish. After incubation for 12 h, the cells were treated with various formulation for 4 h. Then, the cells were co-stained with DCFH-DA (10 μM) and Hoechst (20 μM). After 20 minutes of incubation, the medium was removed and the cells were washed three times with DMEM. The fluorescence imaging of cells was imaged by confocal microscopy.

***In vitro* •OH generation**

GL261 cells were seeded in confocal dish. After incubation for 12 h, the cells were treated with various formulation for 4 h. Then, the cells were co-stained with O26 and Hoechst. After 30 minutes of incubation, the fluorescence imaging of cells was imaged by CLSM.

**Intracellular GSH**

GL261 cells were plated in 6-well plates and incubated for 24 h. Subsequently, the cells were exposed to K-SAN without/with laser irradiation. The GSH contents were measured using a DTNB kit The assay was carried out according to the manufacturer’s instructions. The absorbance of 412 nm was measured by a microplate reader.

**Analysis of the change of mitochondrial membrane potential (MMP)**

To investigate the MMP, GL261 cells were seeded and incubated for 24 h. Subsequently, the cells were exposed to K-SAN without/with laser irradiation. Then the cells were treated according to the JC-1 kit. The fluorescence imaging of cells was analyzed by confocal microscopy.

**Lipid peroxidation (LPO) initiated by K-SAN**

The cellular LPO assay was carried out by using a BODIPY^581/591^-C11 probe. GL261 cells were seeded and incubated for 24 h. Subsequently, the cells were exposed to K-SAN without/with laser irradiation. Then the cells were stained with BODIPY^581/591^-C11 probe and Hoechst for 30 min. The fluorescence imaging of cells was imaged by CLSM.

**Intracellular GPX4 expression**

GL261 cells were incubated with K-SAN for 24 h. Subsequently, the cells were fixed with 1% PFA at 37 ºC for 15 min and then permeabilized with PBS buffer containing 0.2% Triton X-100 at 37 ºC for 10 min. After blocking with PBS buffer containing 0.05% Tween 20 and 10% goat serum at room temperature for 45 min, the cells were incubated with anti-GPX4 antibodies (Alexa Fluor® 640 Conjugate) in a humidified chamber for 1 h. Finally, the fluorescence images were acquired to analyze the expression levels of GPX4. Moreover, the expression of GPX4 was also measured by western bloting assay.

**Analysis of the change of MDA**

To investigate the MDA, GL261 cells were seeded and incubated for 24 h. Subsequently, the cells were exposed to K-SAN without/with laser irradiation. Then the cells were treated according to the MDA kit. The fluorescence imaging of cells was analyzed by confocal microscopy.

**Analysis of the change of 4-HNE**

To investigate the 4-HNE, GL261 cells were seeded and incubated for 24 h. Subsequently, the cells were exposed to K-SAN without/with laser irradiation. Then the cells were treated according to the 4-HNE kit. The fluorescence imaging of cells was analyzed by confocal microscopy.

**Enzyme-linked immunosorbent assay**

Commercially available enzyme-linked immunosorbent assay (ELISA) kits were used to measure the leves of 4-hydroxynonenal (4-HNE) in the indicated samples according to the manufacturers’ instructions.

***In vivo* antitumor efficacy**

Animal experiments were performed according to the protocol approved by The Ethical Committee of Fujian Medical University. The right hind legs of all mice were subcutaneously transplanted with GL261 cells (1 × 10^6^ cells suspended in 100 μL of PBS). The tumor-bearing mice were used for antitumor treatment until the tumor volume reached about 70 mm^3^. Tumor volume = (tumor length) × (tumor width)^2^/2.

GL261 tumor-bearing nude mice were randomly divided into four groups (6 mice per group) and intravenously administrated with 5 mg/kg K-SAN or PBS. Living imaging was performed. After 24 h post-injection, the two laser exposure groups were irradiated (1 W/cm^2^, 808 nm) for 5 min. The surface temperature of the mice was also recorded. The orbital blood of one mouse in each group was collected for biochemical and blood routine indexes measurement. The tumor volumes and body weights were recorded every two days. After 16 days of treatment, one mice from each group were euthanatized for histological examination. Sacrificed mice were not included in the statistical results.

**Statistical analysis**

All quantitative data were expressed as the mean ± standard deviation (SD). Statistical analyses were performed using the Student’s two-tailed t-test (*P < 0.05, **P < 0.01, ***P < 0.001, ****P< 0.0001).


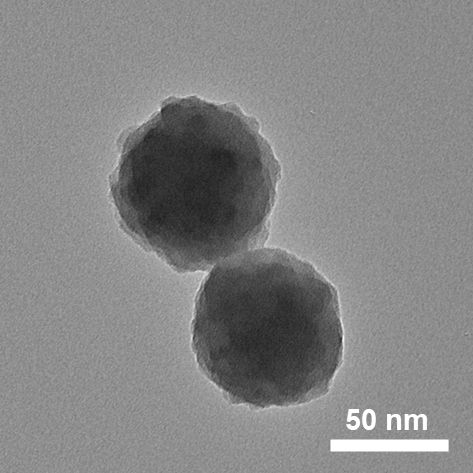


**Figure S1.** The TEM images of PDA.


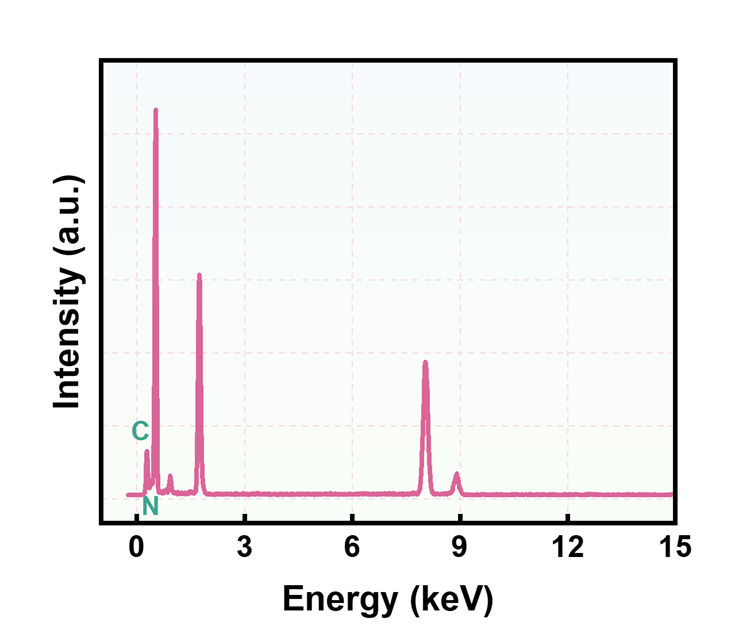


**Figure S2.** The EDX spectrum of K-SAN.


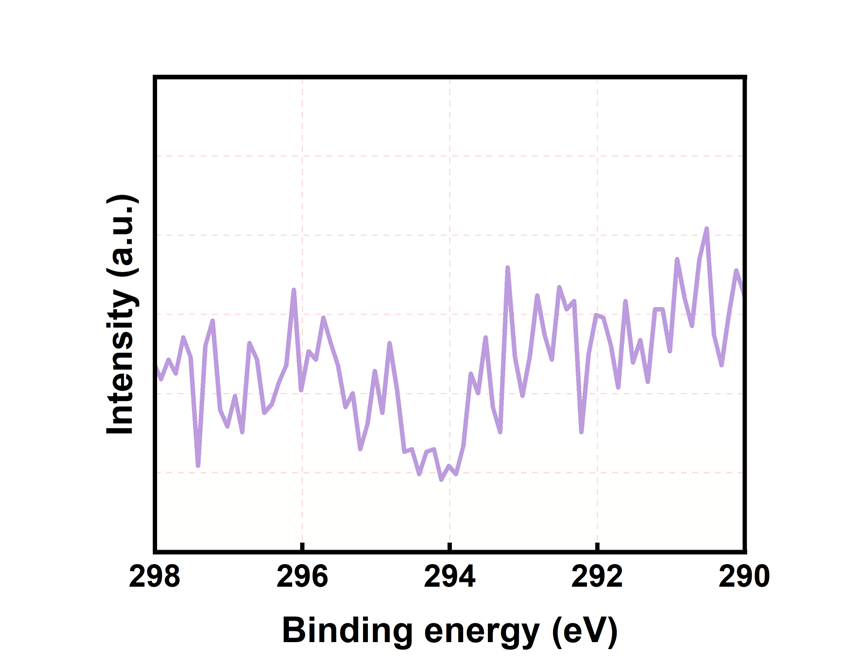


**Figure S3.** The K 2p XPS of K-SAN.

**Table S1.** EXAFS fitting parameters of various samples at the k K-edge (S0^2^ = 0.9217).

| **Sample** | **Path** | ***CN^a^*** | ***R*(Å)*^b^*** | ***σ*^2^ (Å^2^)*^c^*** | **Δ*E*_0_(eV)*^d^*** | ***R* factor** |
| --- | --- | --- | --- | --- | --- | --- |
| **K K-edge (*Ѕ*_0_^2^=0.917)** | | | | | | |
| **K1** | **K-N** | **4.0±0.4** | **2.766±0.032** | **0.0261** | **-8.7±2.0** | **0.0110** |


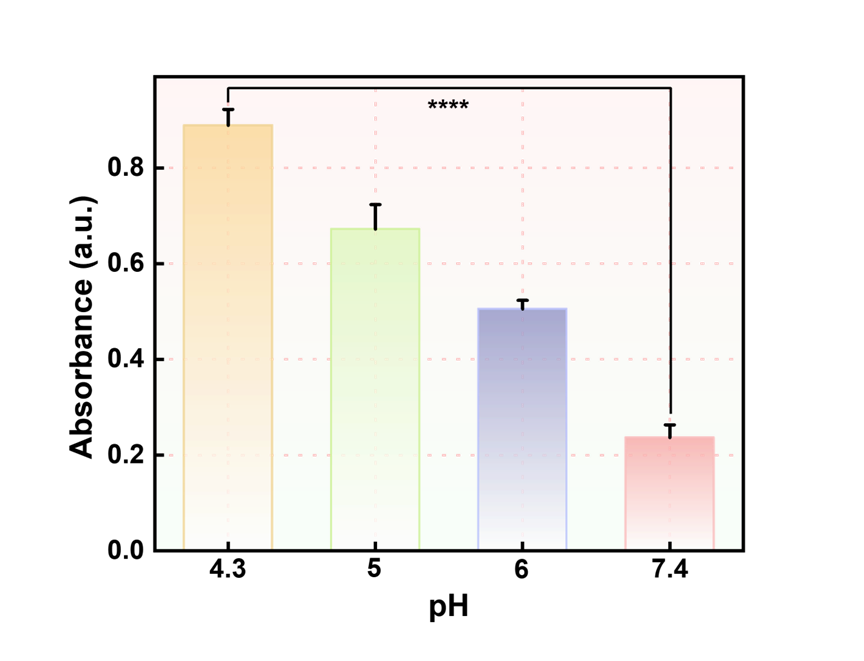


**Figure S4.** TMB assay for measuring POD-like activity of the K-SAN at the different pH. The absorbance of the produced ox-TMB at 652 nm was measured by the Bio-Rad 680 microplate reader after incubating K-SAN with 40 μg /mL TMB for 5 min.


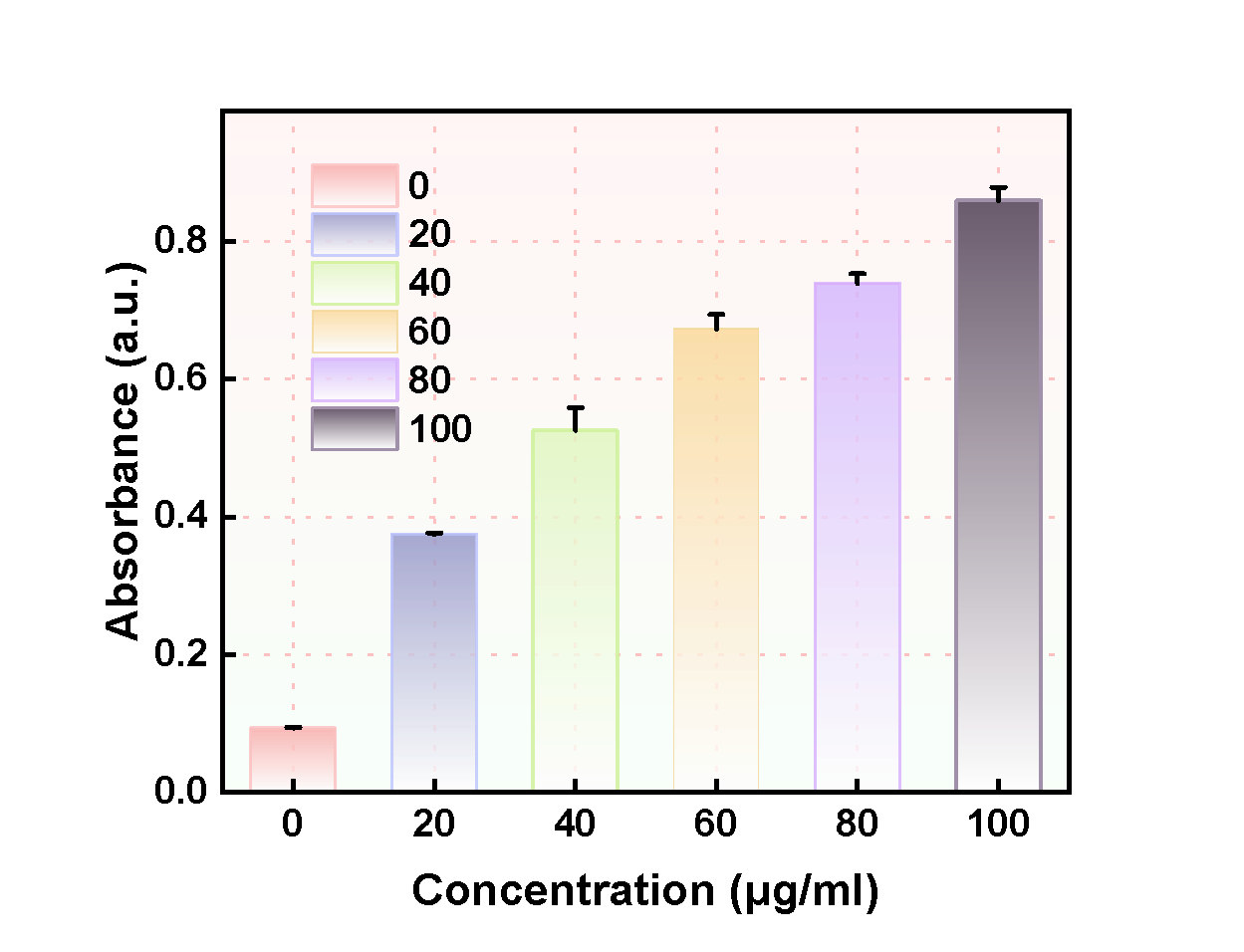


**Figure S5.** The absorbance of the produced oxide at 652 nm was measured by the Bio-Rad 680 microplate reader after incubating the different concentrations of K-SAN with 40 μg /mL TMB for 5 min.


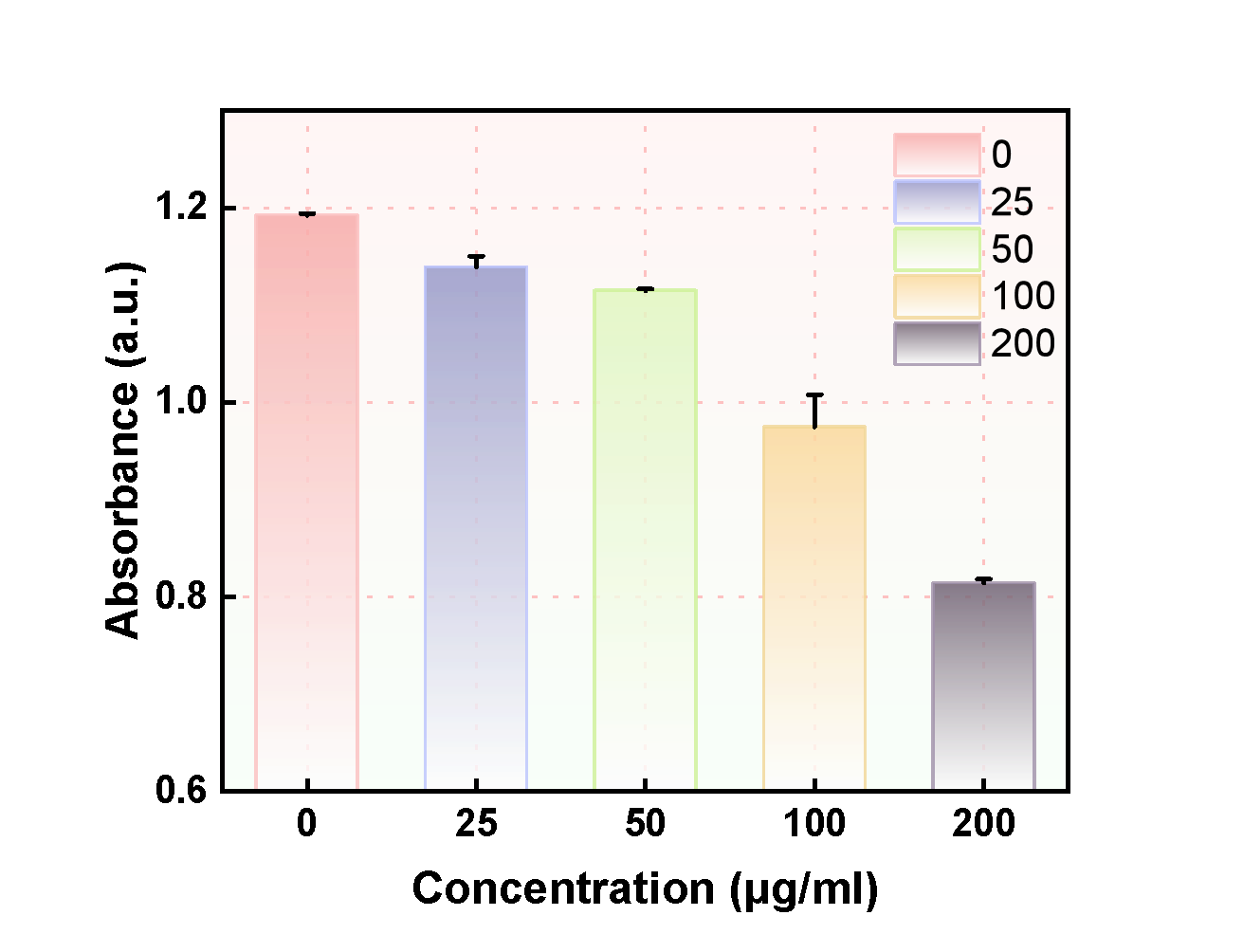


**Figure S6.** The absorbance of the produced oxide at 680 nm was measured by the Bio-Rad 680 microplate reader after incubating the different concentrations of K-SAN with MB for 5 min.

**
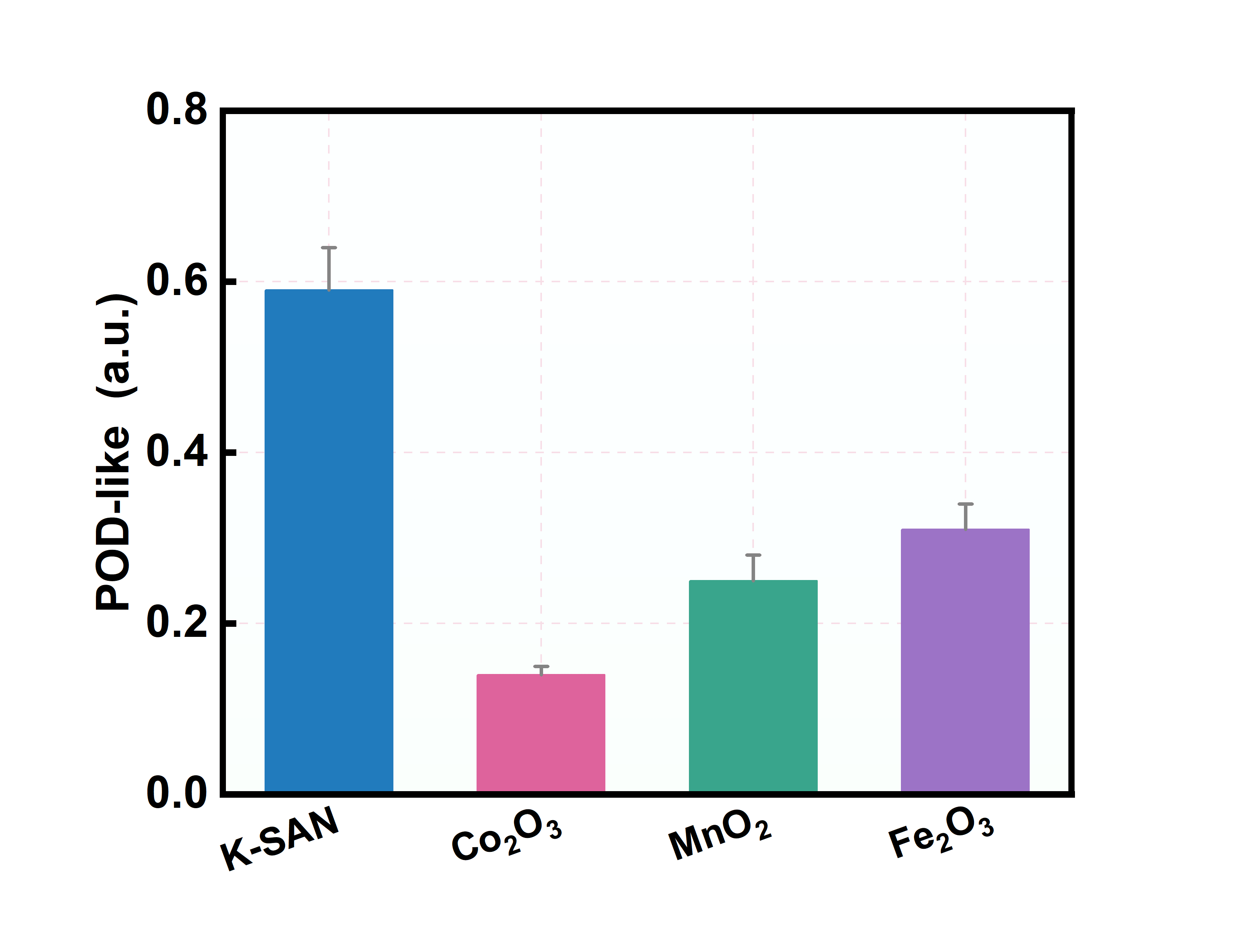
**

**Figure S7.** The POD-like activity of different nanozymes based on TMB assay.


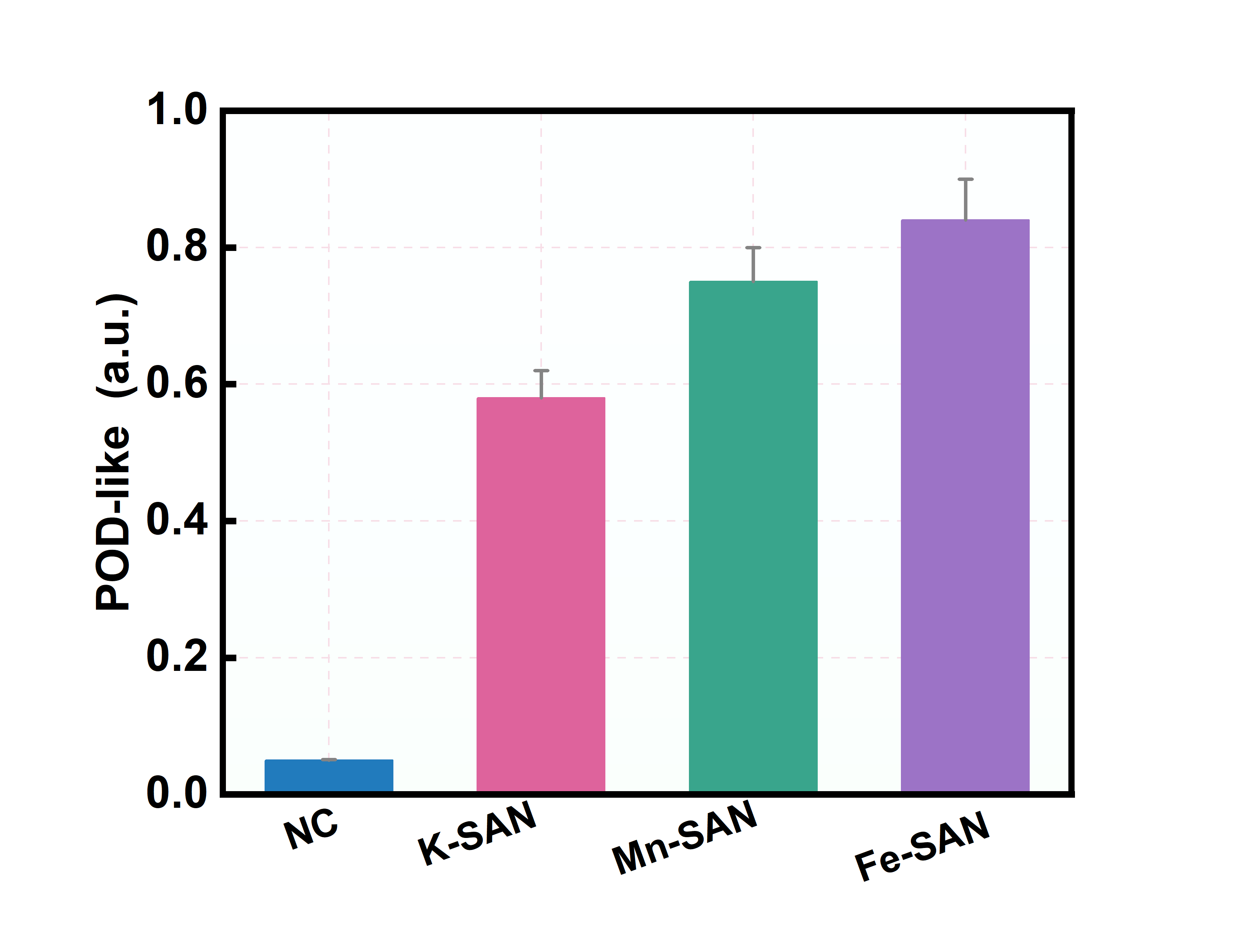


**Figure S8.** The POD-like activity of different SANs based on TMB assay (NC: nitrogen doped carbon).


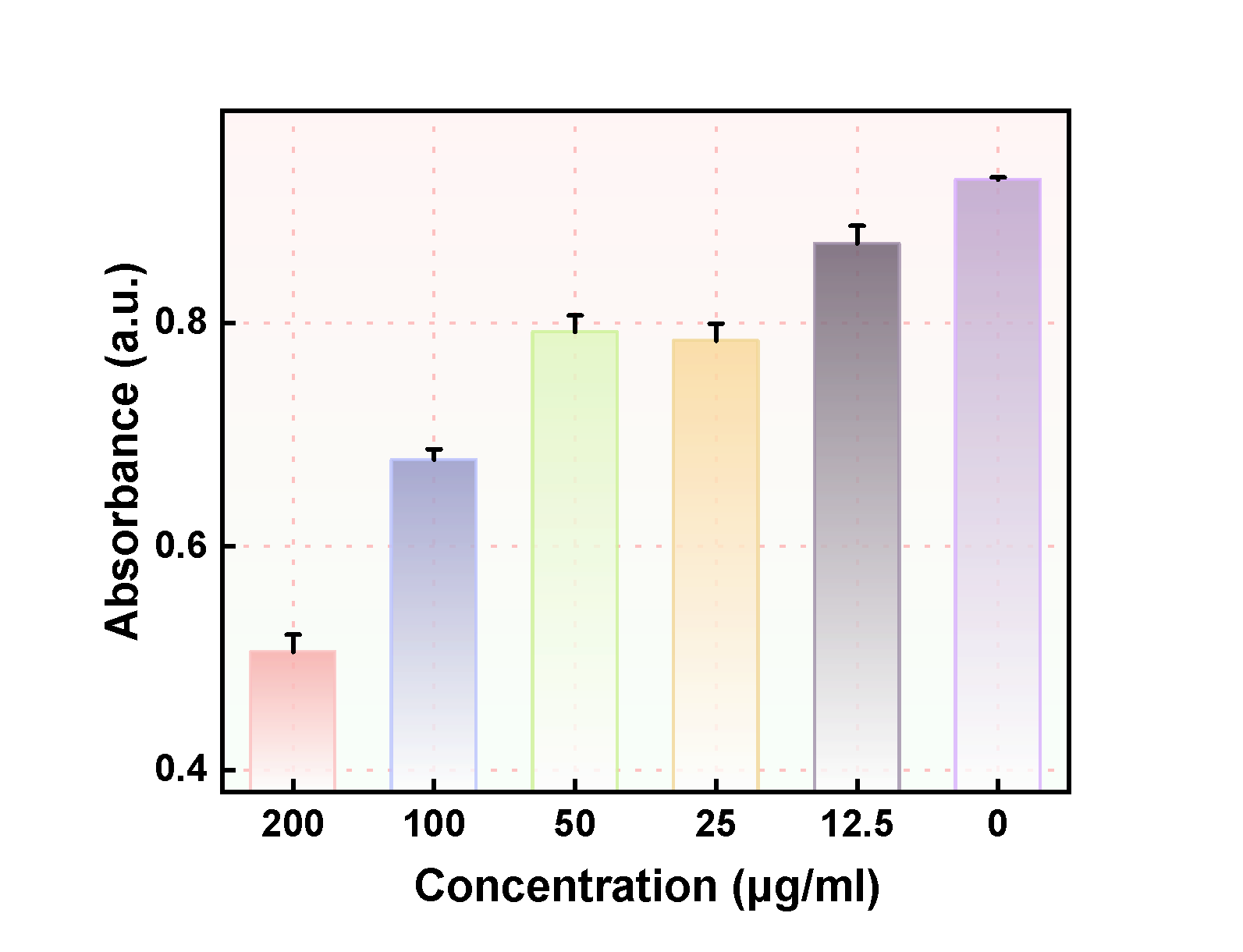


**Figure S9.** The absorbance of the produced oxide at 412 nm was measured by the Bio-Rad 680 microplate reader after DTNB incubated with K-SAN in the presence of GSH.


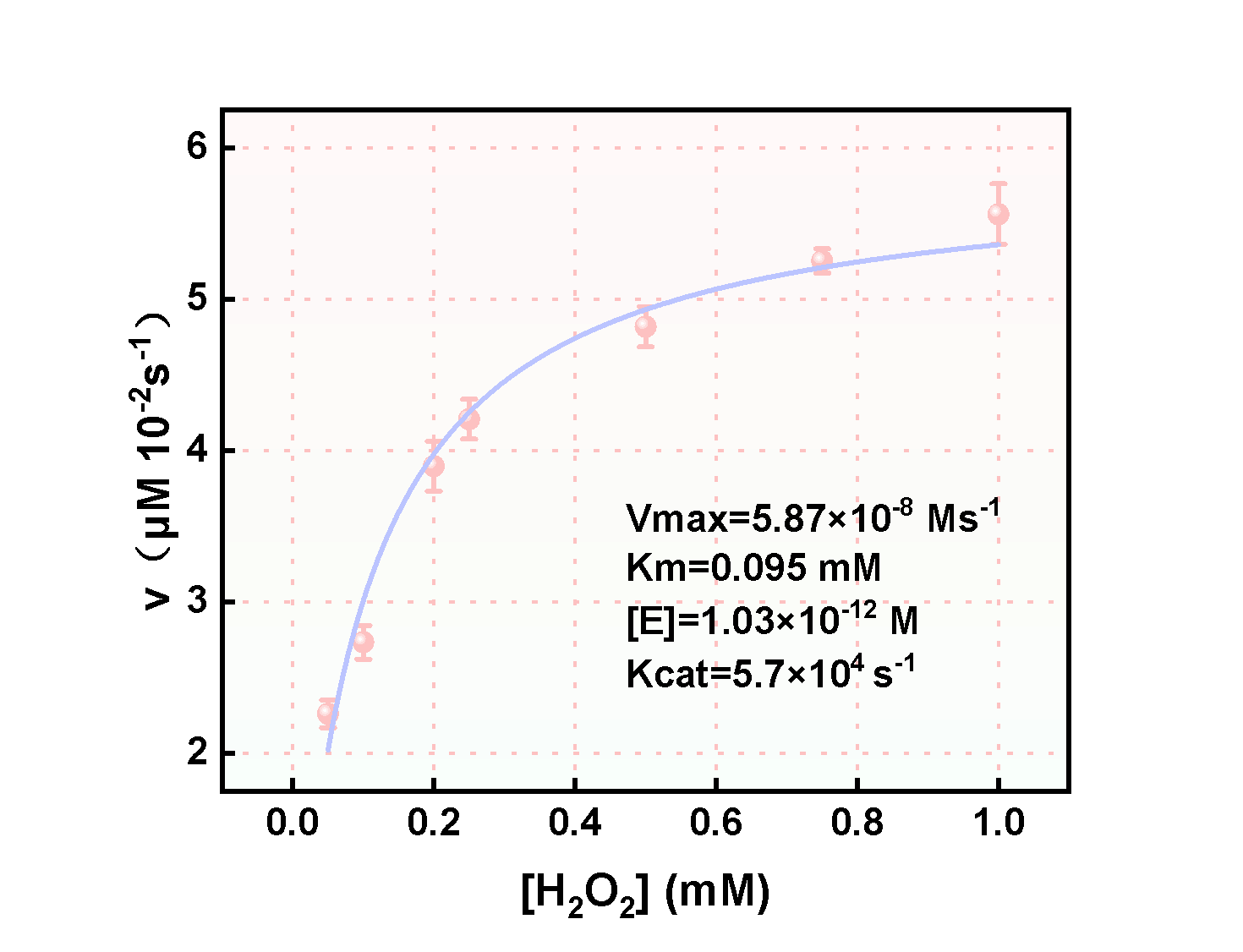


**Figure S10**. (a) Steady-state kinetic assay of the K-SAN using H_2_O_2_ as the substrate. 40 μg/mL K-SAN, pH = 5.0, 40 μg/mL TMB.


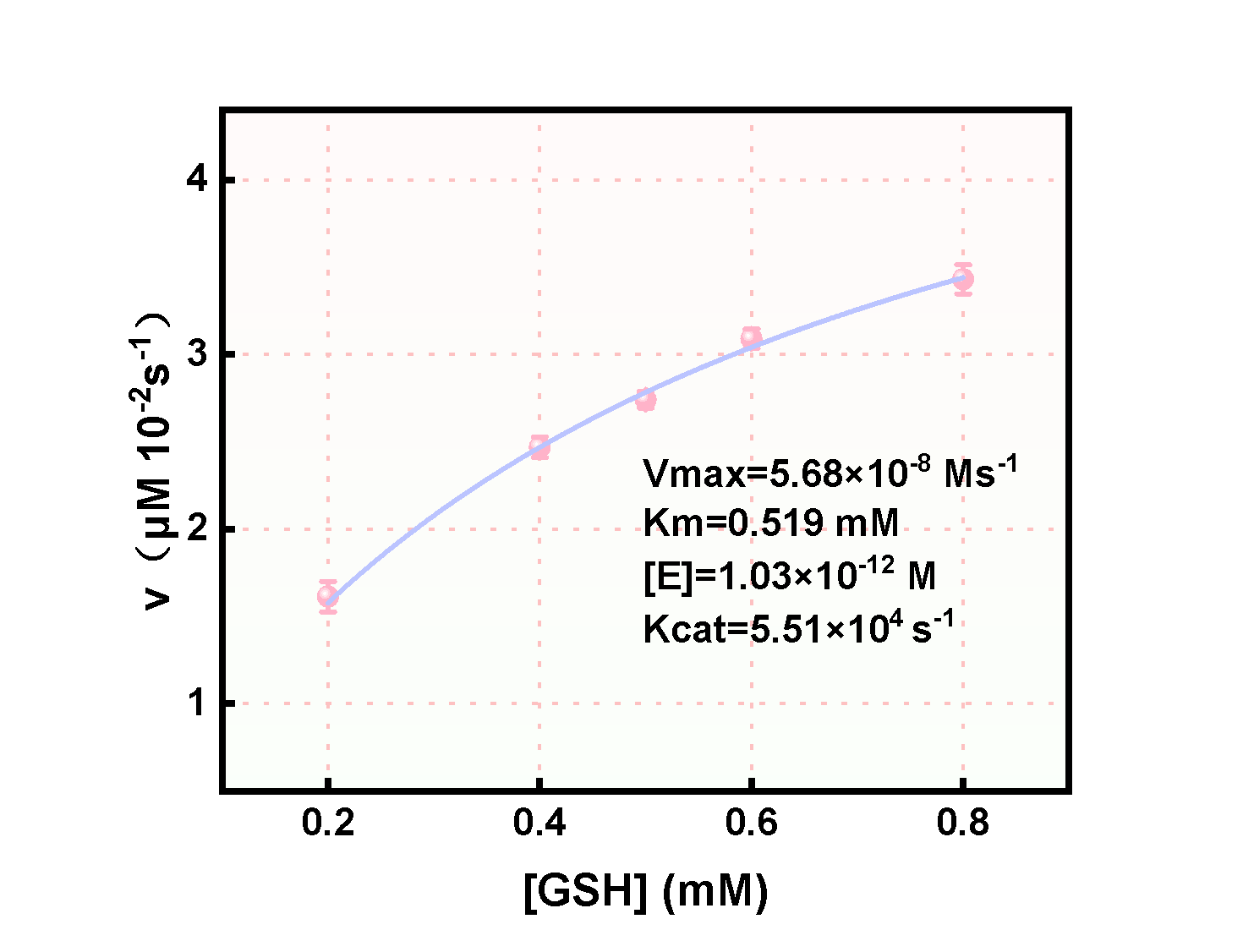


**Figure S11**. (a) Steady-state kinetic assay of the K-SAN using GSH as the substrate. 40 μg/mL K-SAN, pH = 5.0, 50 μg/mL DTNB.


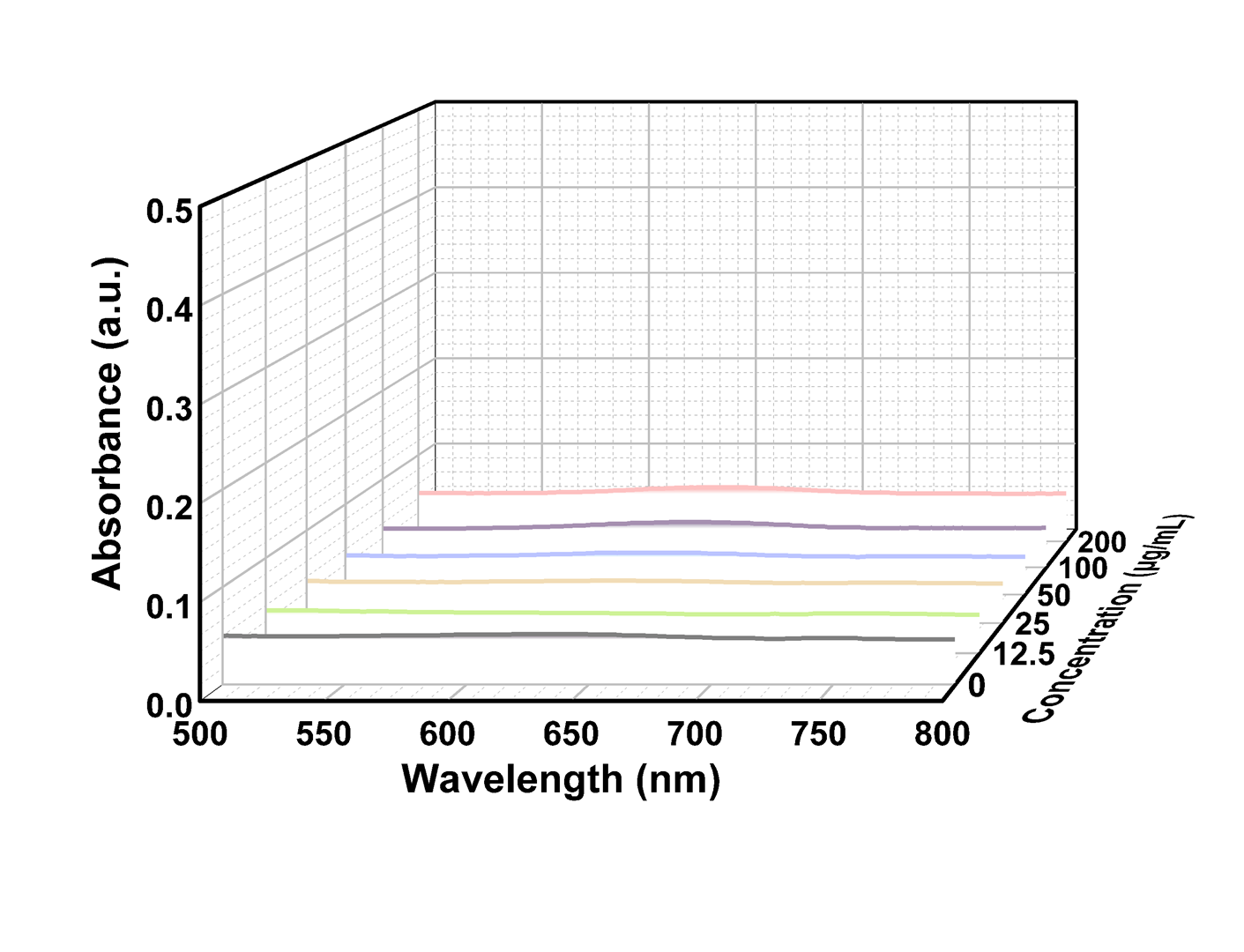


**Figure S12**. POD-like activity of the control sample (NC) without potassium doping.


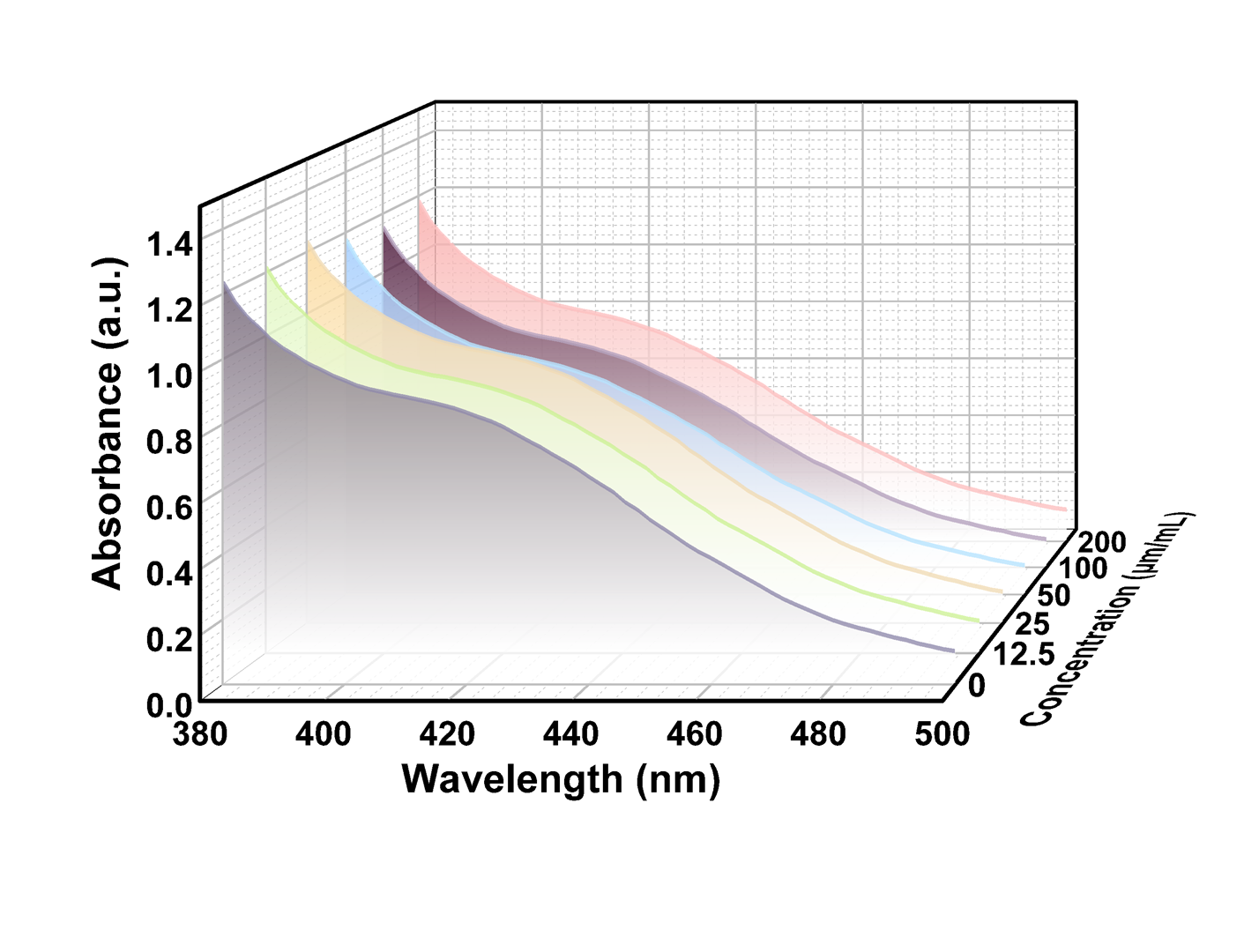


**Figure S13**. GSHOx-like enzyme activity of the control sample without potassium doping.


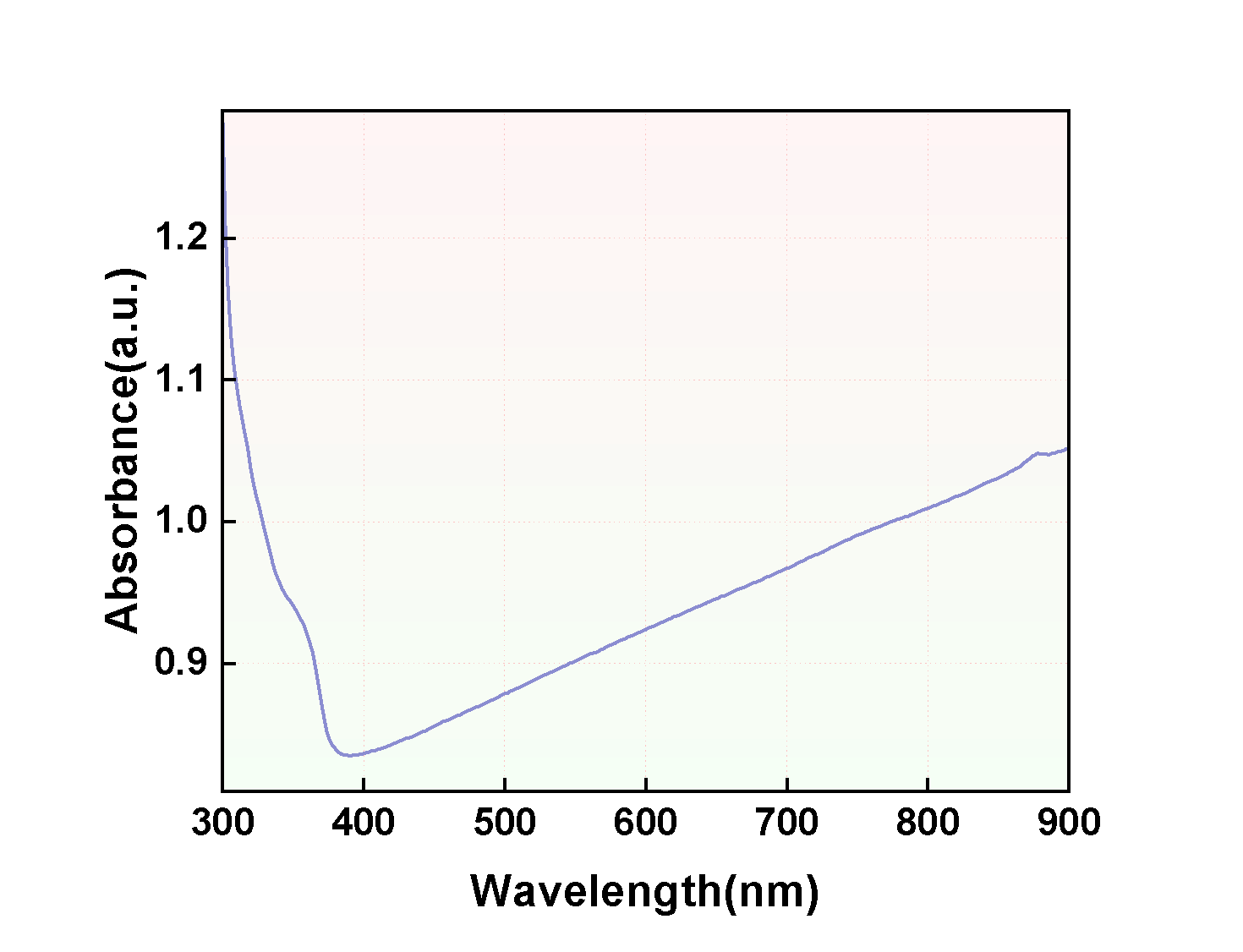


**Figure S14.** Absorbance of K-SAN under various wavelength illuminations.


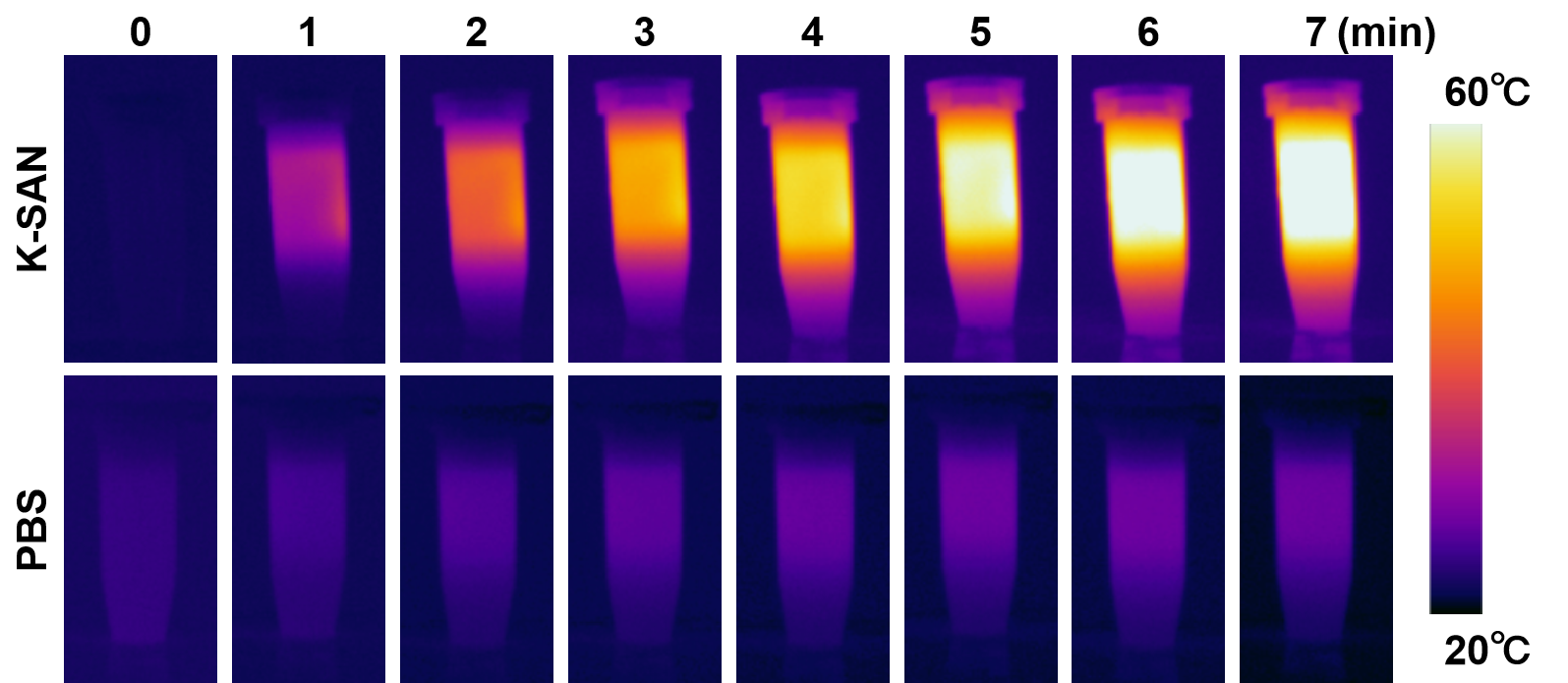


**Figure S15.** The infrared thermal (IT) imaging comparison of K-SAN under 808nm laser irradiation and in the dark environment.


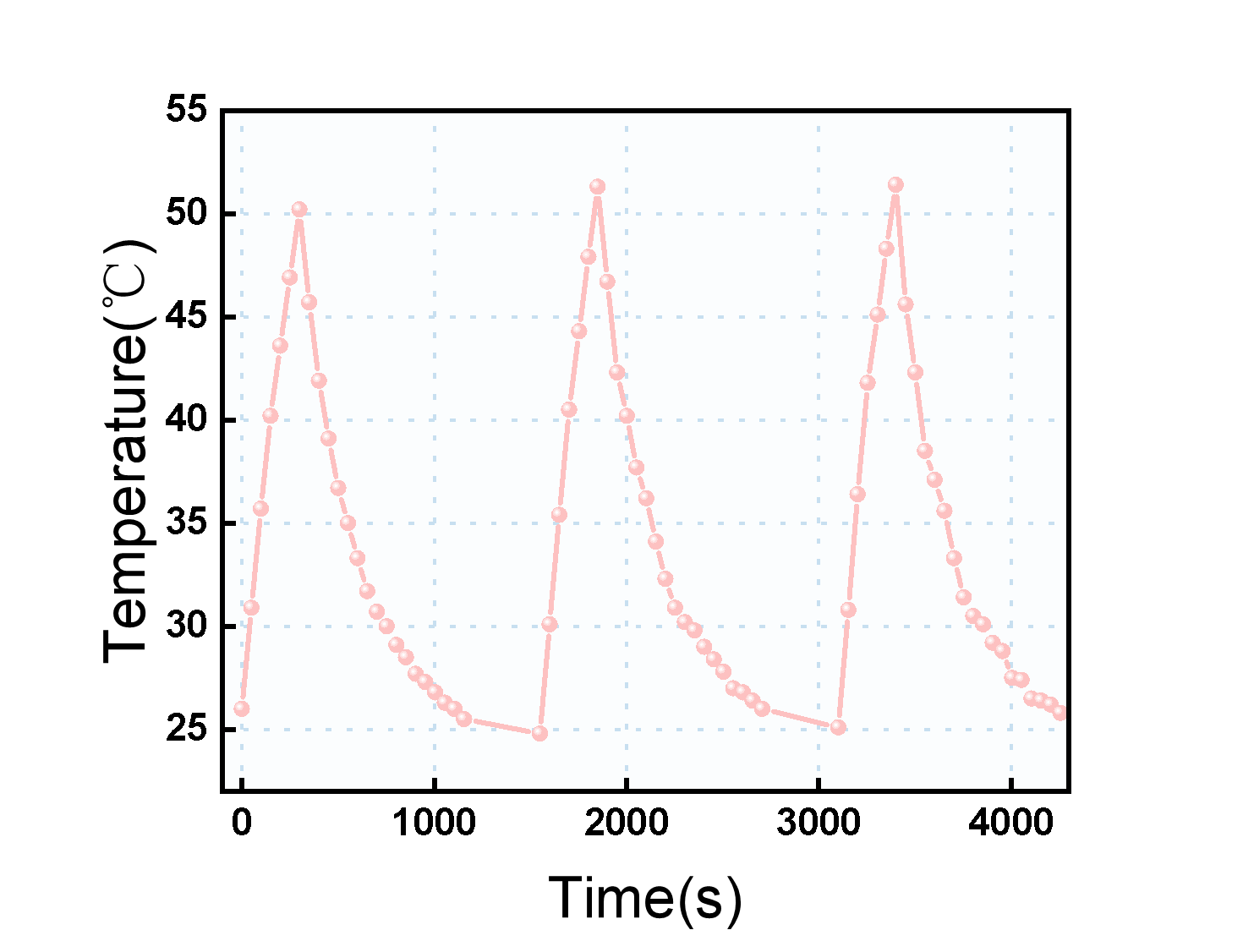


**Figure S16.** Repeated three cycles to investigate the photothermal stability of the material.


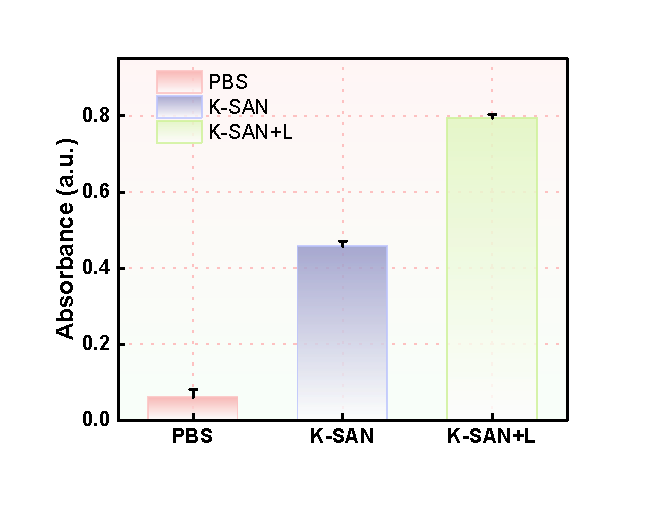


**Figure S17.** The absorbance of the produced oxide at 652 nm was measured by the Bio-Rad 680 microplate reader after TMB incubated with K-SAN plus laser in the presence of H_2_O_2_.


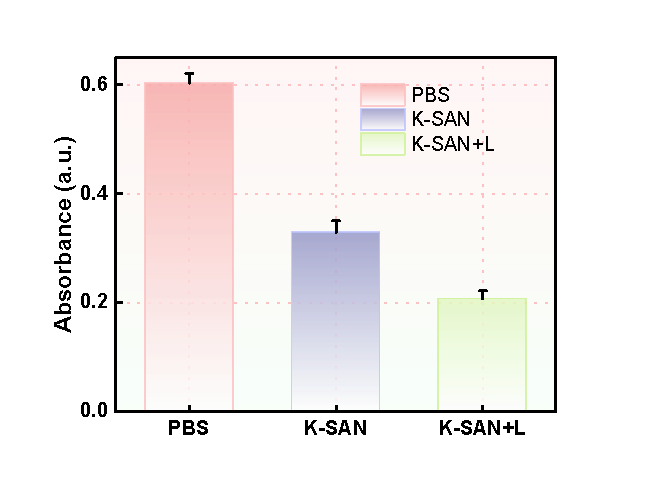


**Figure S18.** The absorbance of the produced oxide at 412 nm was measured by the Bio-Rad 680 microplate reader after DTNB incubated with K-SAN plus laser in the presence of GSH.


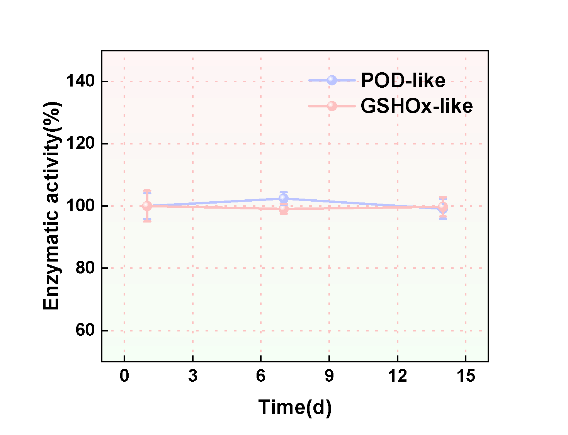


**Figure S19.** The POD-like and GSHOx-like activities of K-SAN as a function of incubation time.


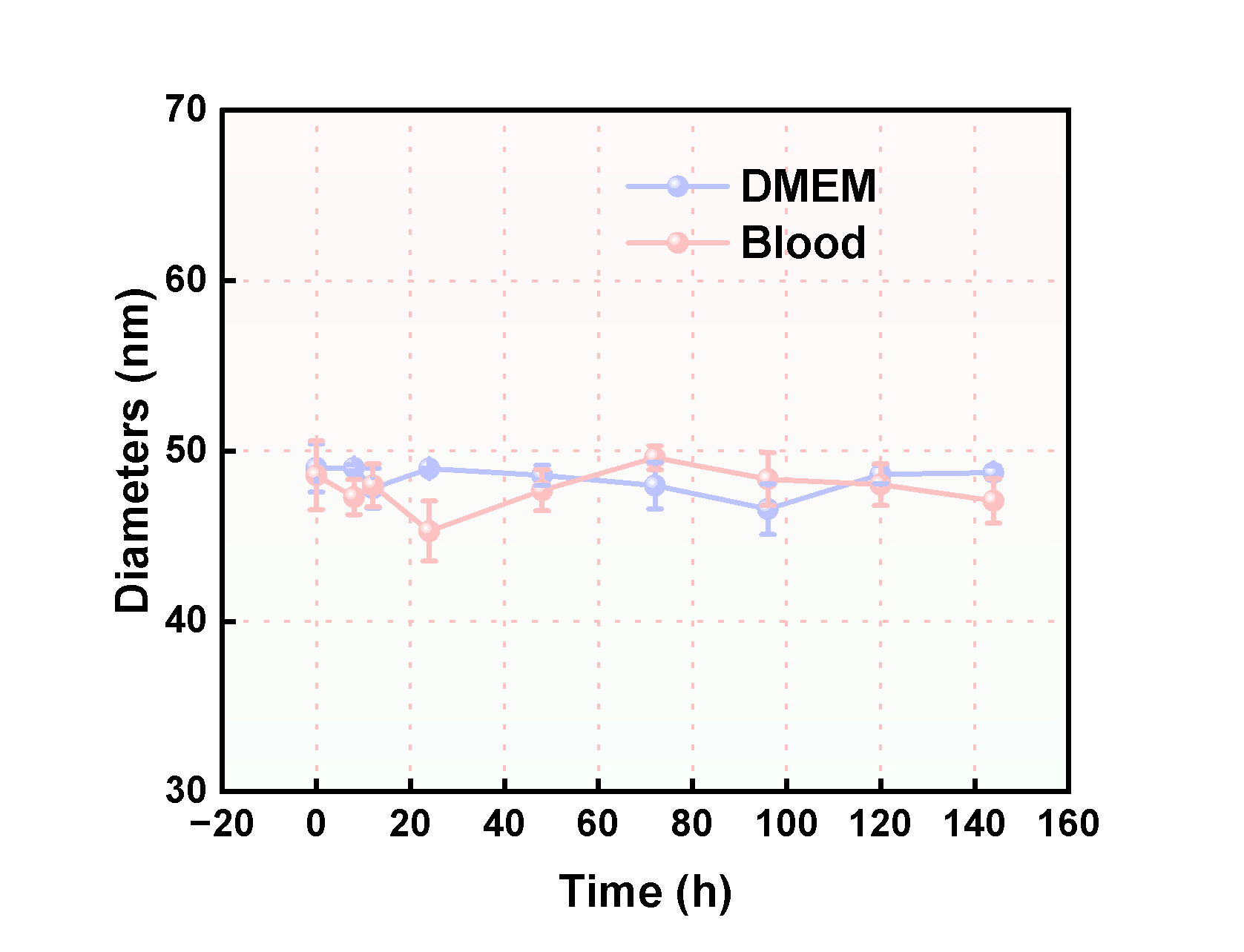
 **Figure S20.** The long-term stability of K-SAN in DMEM and blood.


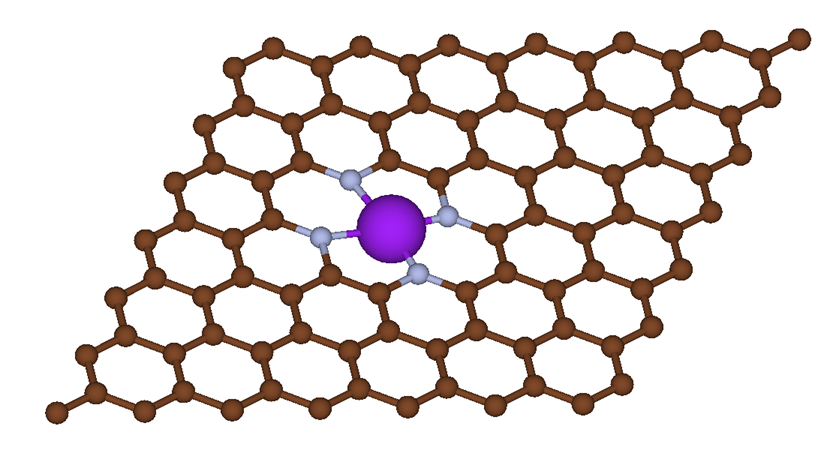


**Figure S21.** Geometrically optimized structures of K-SAN.


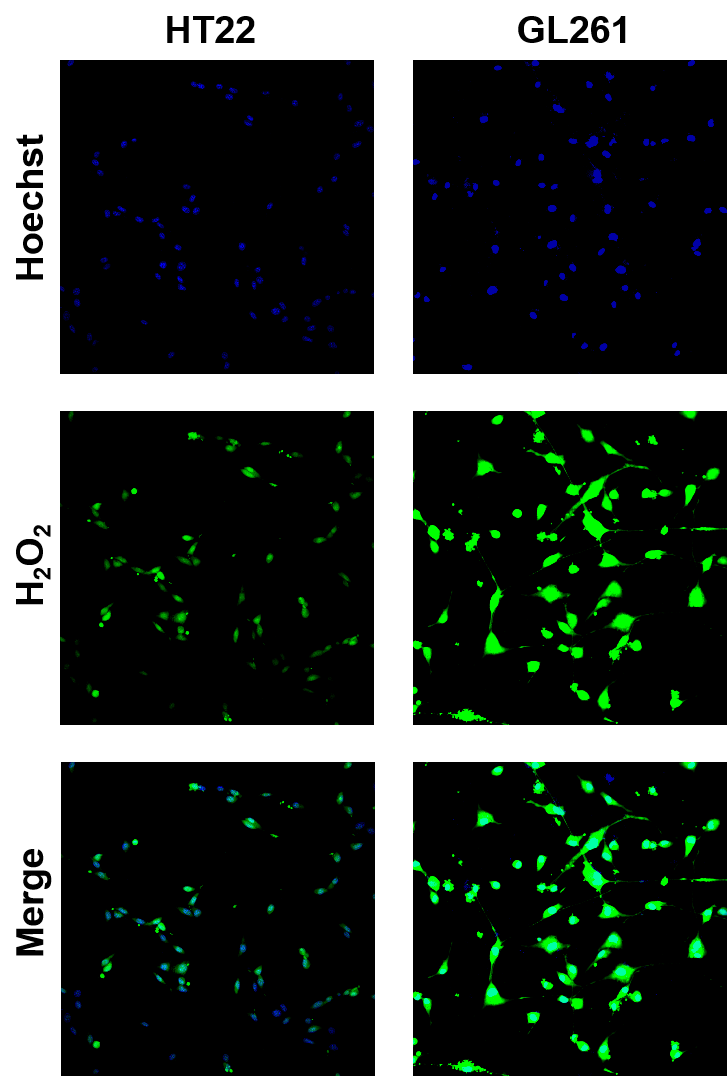


**Figure S22.** Hydrogen peroxide content in HT22 and GL261


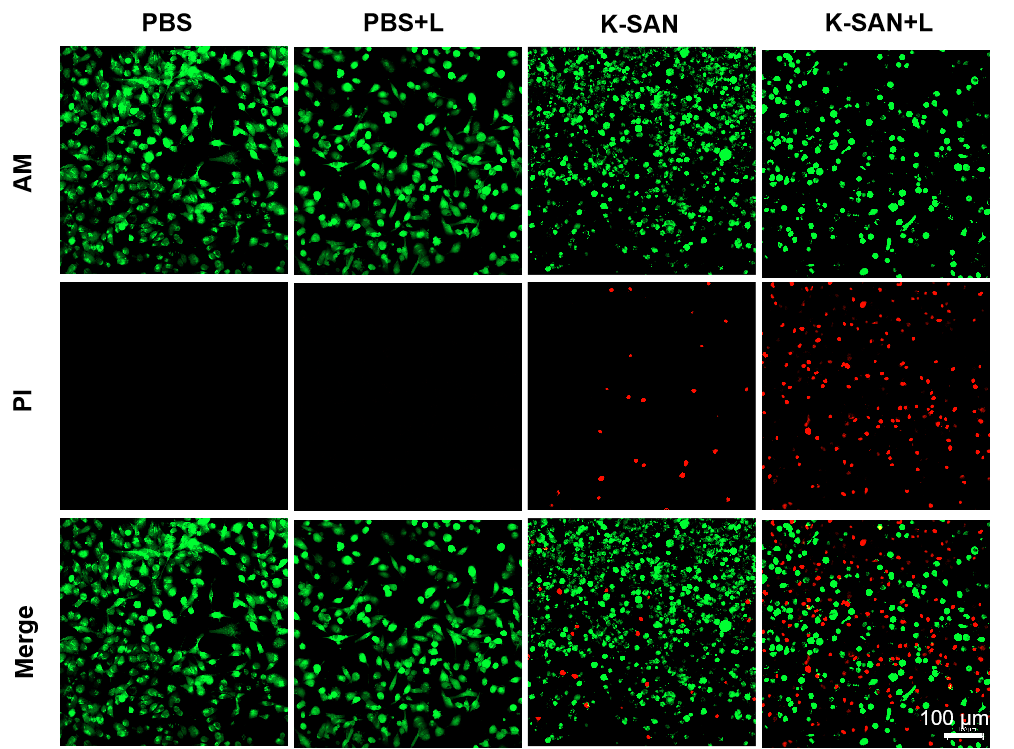


**Figure S23.** Calcein-AM/PIco-stained GL261 cells incubated with various formulation.


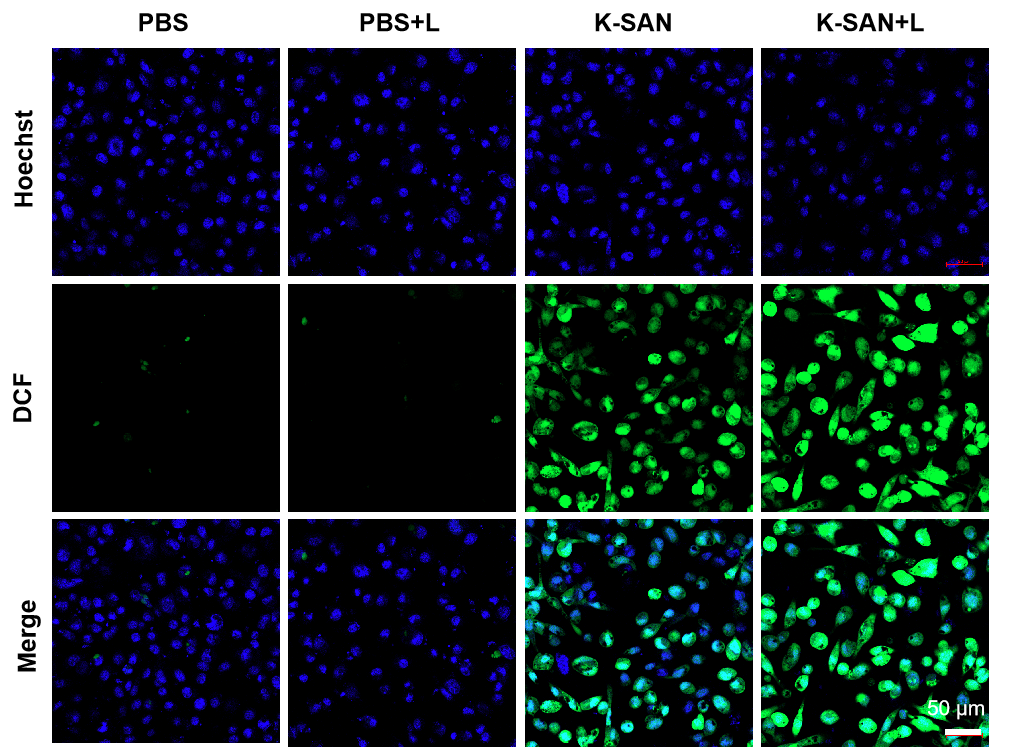


**Figure S24.** DCF fluorescence images of GL261 cells exposed to various formulations.


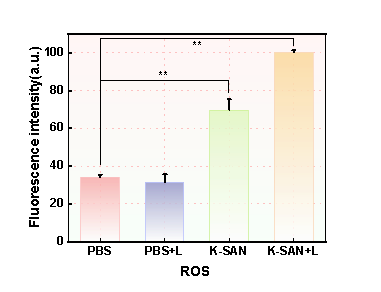


**Figure S25.** DCF fluorescence images associated quantification of GL261 cells exposed to various formulations.


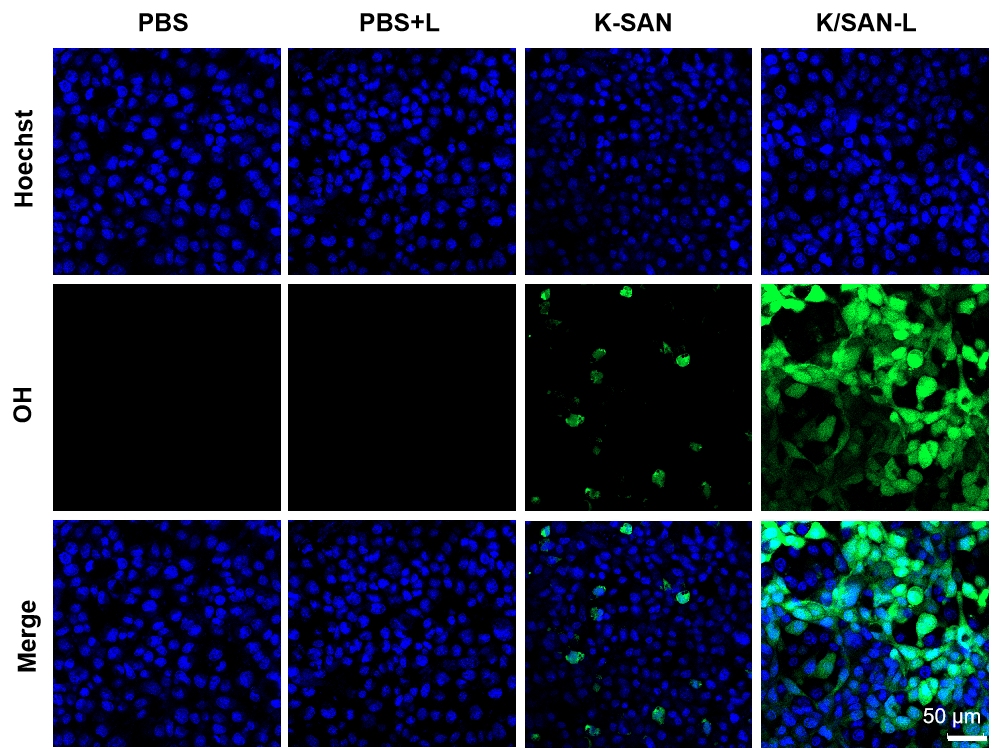


**Figure S26.** CLSM images of •OH probe O26-stained GL261 cells exposed to various formulations.


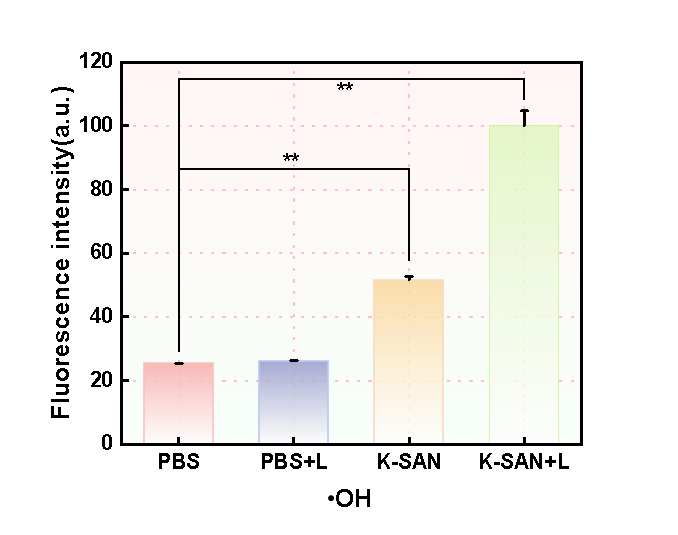


**Figure S27.** •OH probe O26-stained fluorescence images associated quantification of GL261 cells exposed to various formulations.


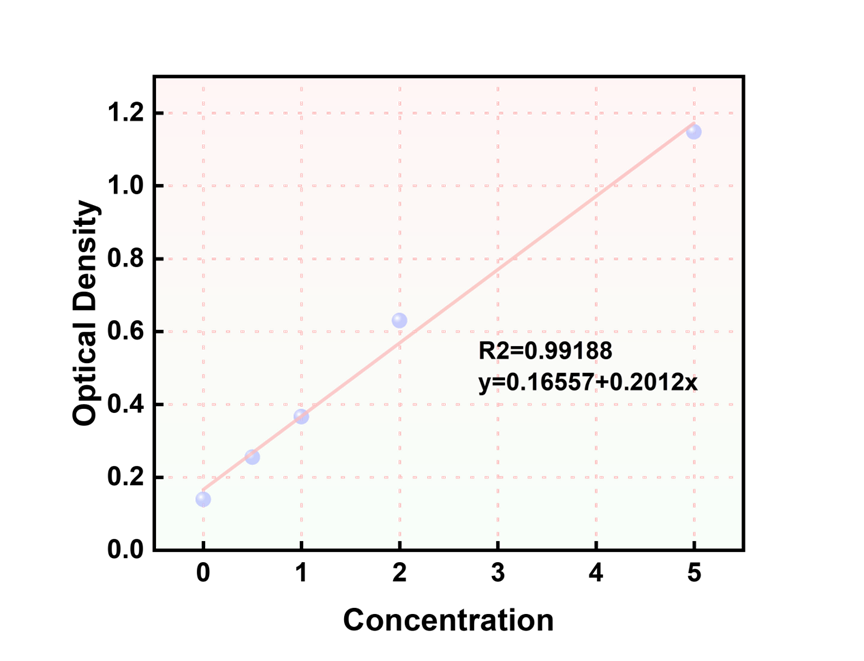


**Figure S28.** The standard concentration-absorbance curves of GSH in GL261 cells.


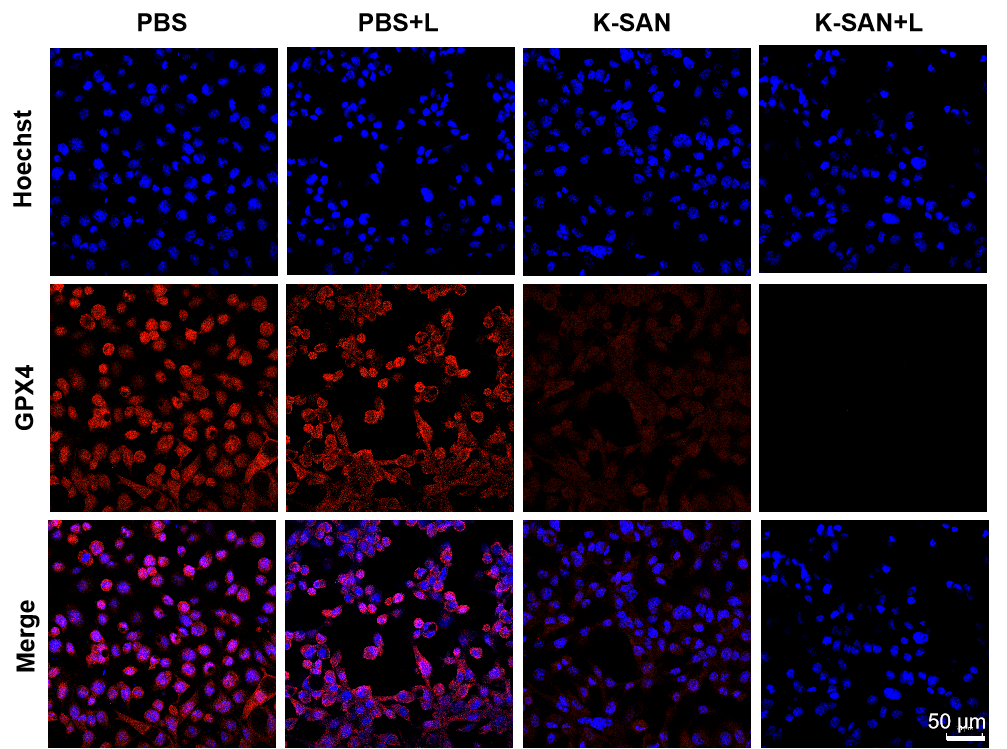


**Figure S29.** CLSM images of GPX4 expression in GL261 cells treated with different formulations.


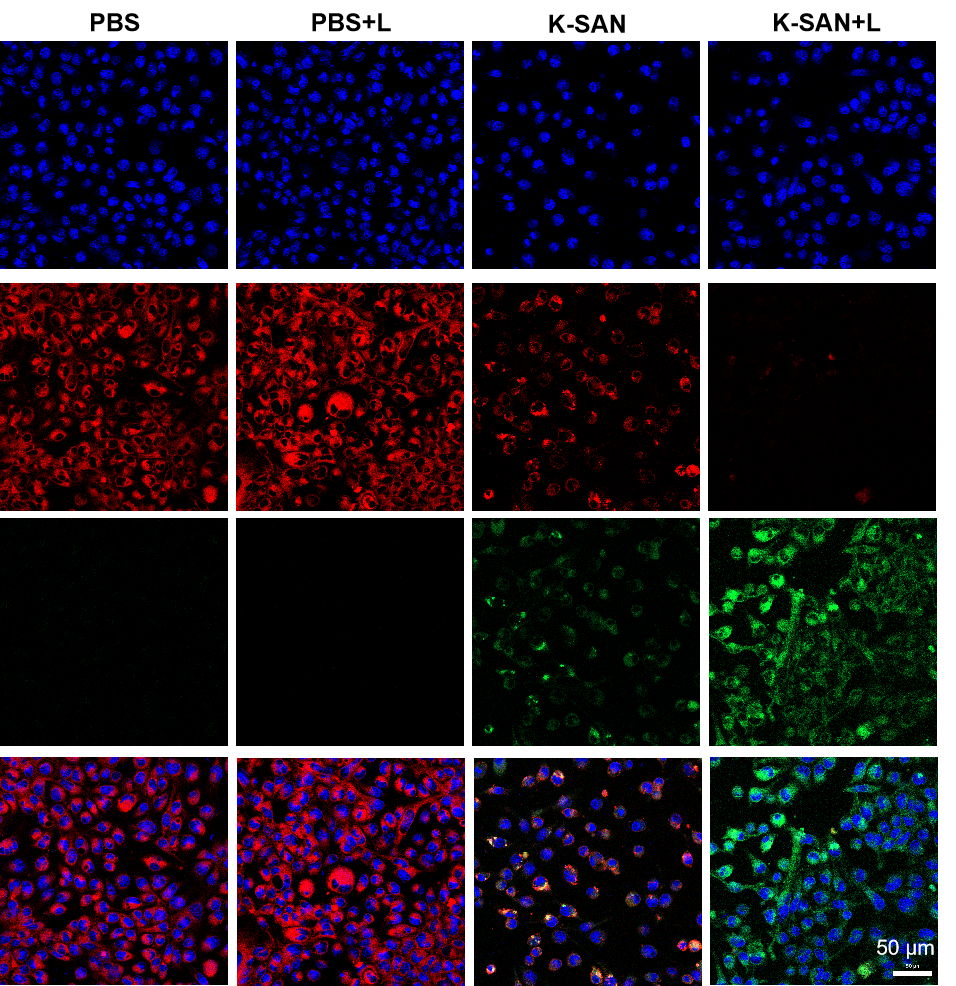


**Figure S30.** CLSM images of MDA expression in GL261 cells treated with different formulations.


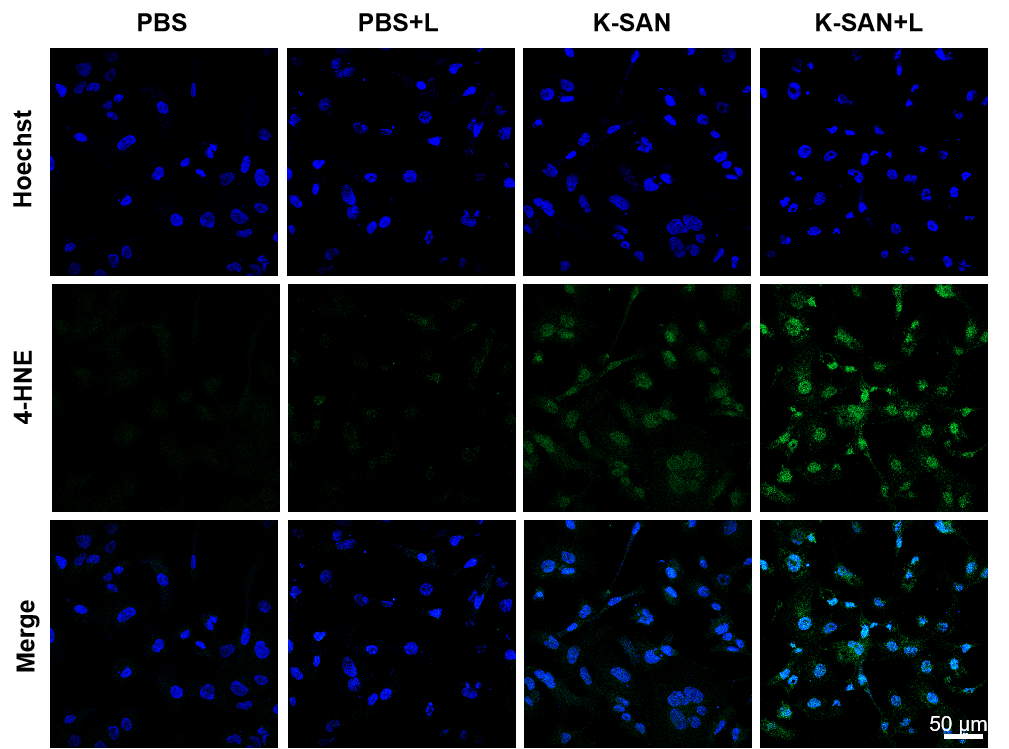


**Figure S31.** CLSM images of 4-HNE expression in GL261 cells treated with different formulations.


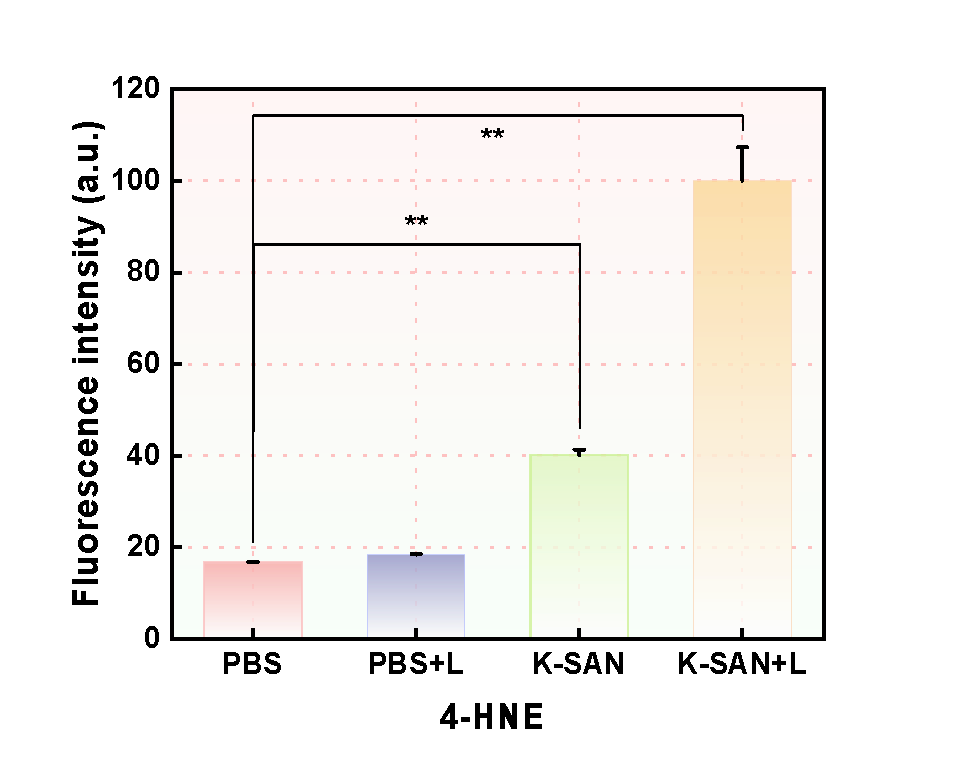


**Figure S32.** 4-HNE fluorescence images associated quantification of GL261 cells exposed to various formulations.


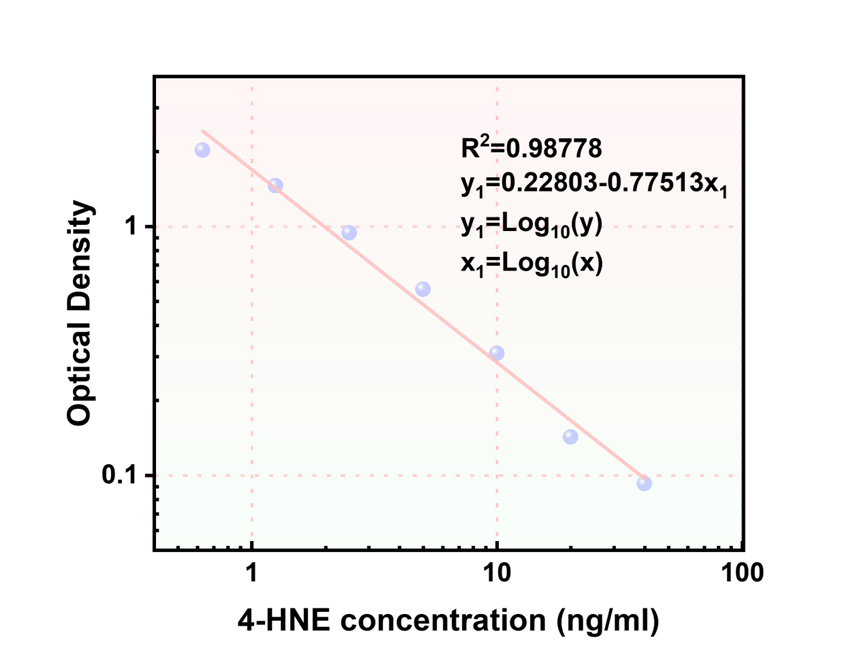


**Figure S33.** The standard concentration-absorbance curves of 4-HNE in GL261 cells.
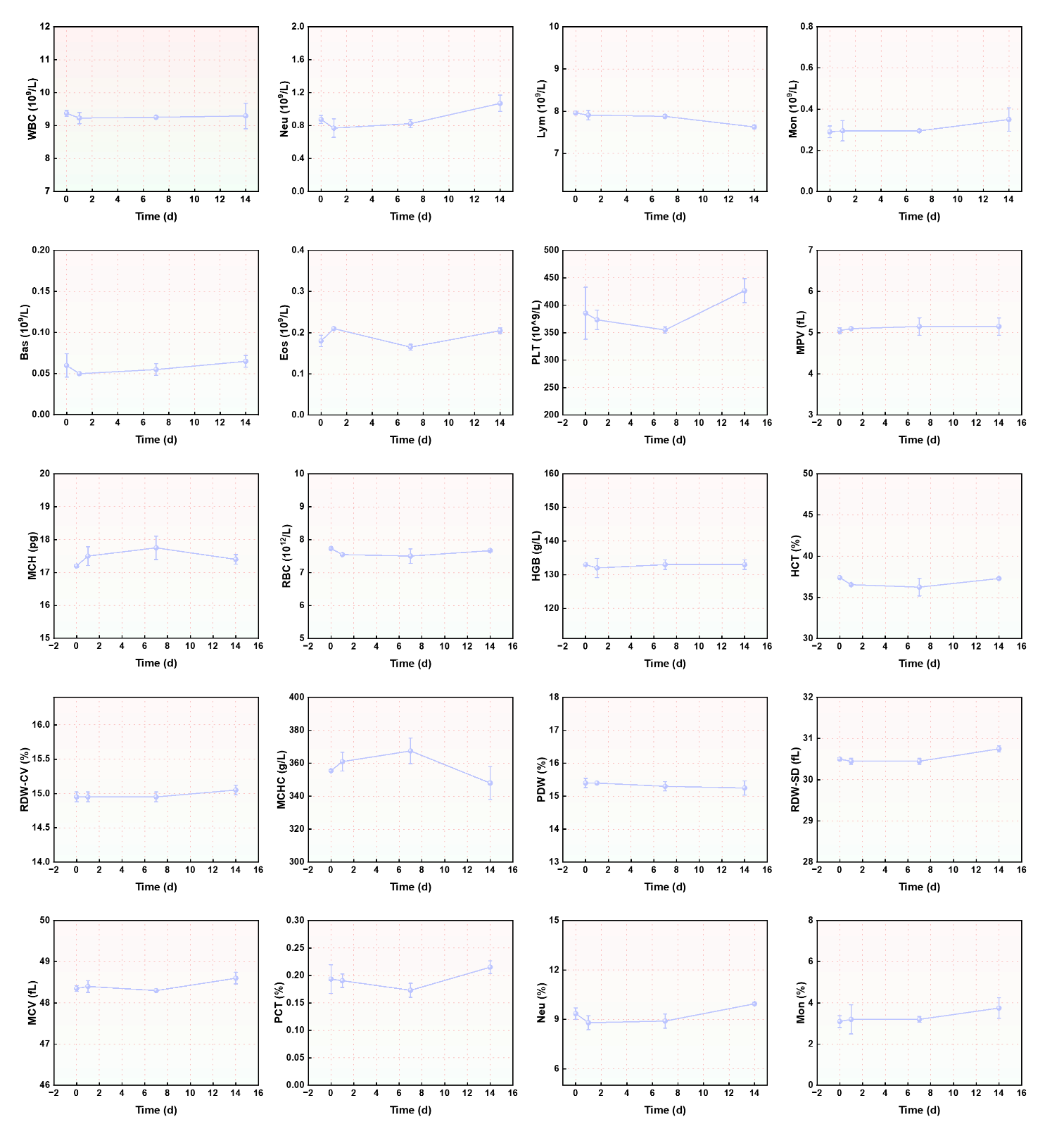


**Figure S34.** Routine blood examination in nude mice at days 0, 1, 7, and 14 after injecting with K-SAN. (White blood cell, WBC\ Neutrophil, Neu\ Lymphocyte, Lym\ Monocyte, Mon\ Basophil, Bas\ Eosinophil, Eos\ Platelet, PLT\ Mean platelet volume, MPV\ Mean corpuscular hemoglobin, MCH\ Red blood cell, RBC\ Hemoglobin, HGB\ Hematocrit, HCT\ Red blood cell coefficient of variation\ Mean corpuscular hemoglobin concentration, MCHC\ Platelet distribution width, PDW\ Red blood cell distribution width\ Mean corpuscular volume, MCV\ Plateletocrit, PCT\ Neu percentage\ Mon percentage)


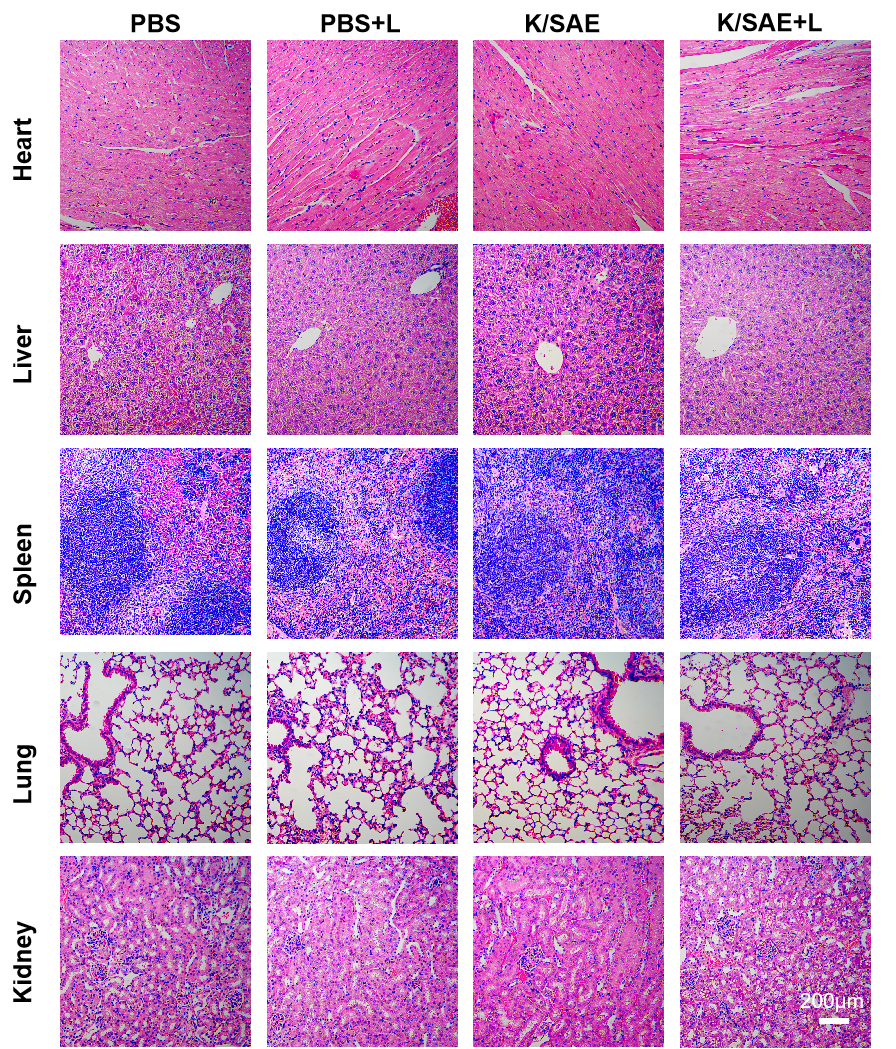


**Figure S35.** Hematoxylin and eosin (H&E)-stained images of major organs harvested from different groups of mice at 16 days post-treatment.


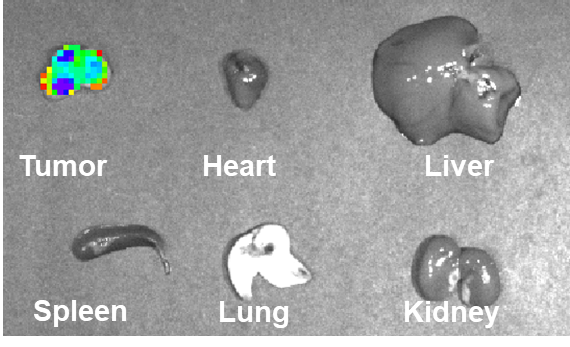


**Figure S36.** Fluorescence images of the major organs (Tumor, heart, liver, spleen, lung, kidney) and tumor collected from the tumor bearing mice at 24 h after intravenous injection.


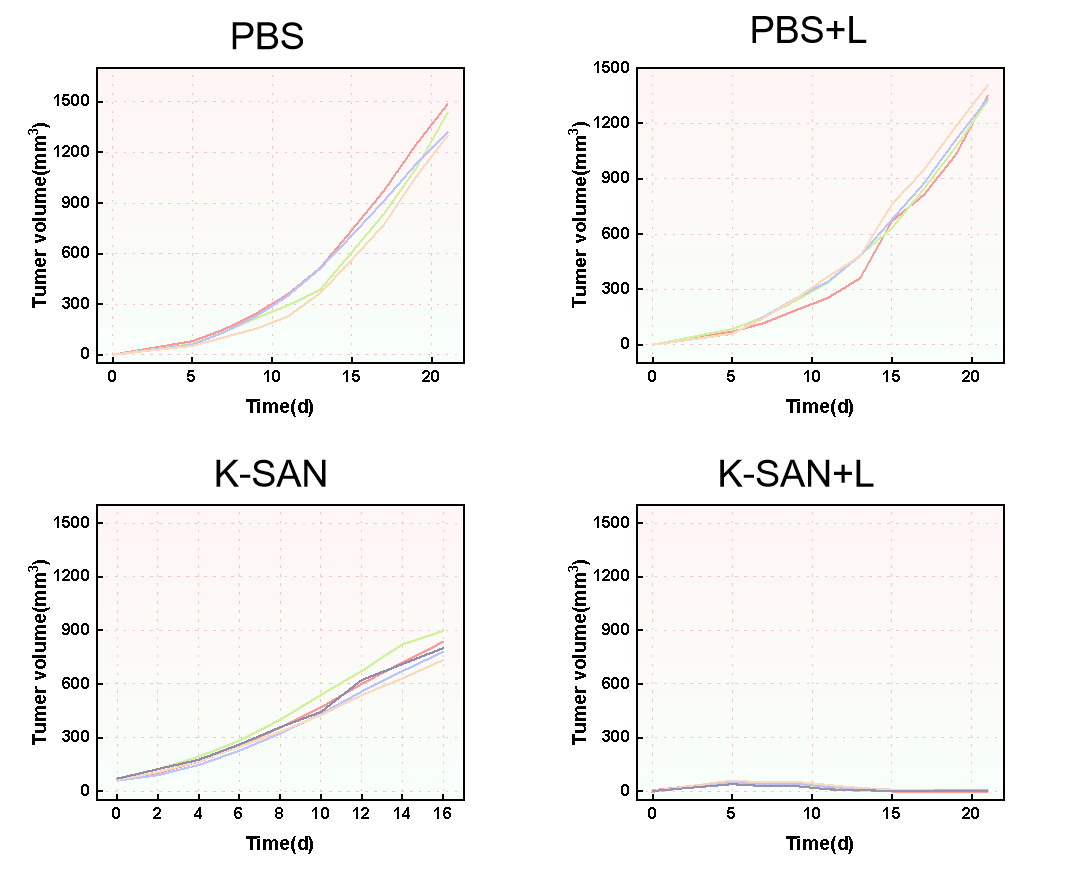
 **Figure S37.** The tumor growth curves of GL261 tumor-bearing mice following varying treatments.


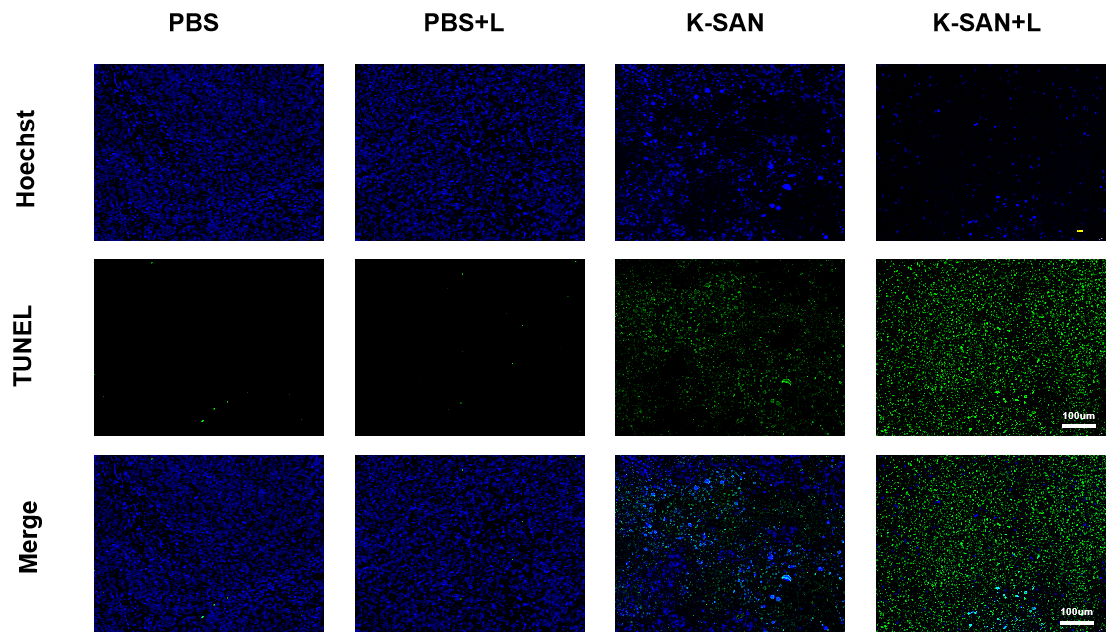


**Figure S38.** TUNEL staining of tumor slices from different groups.


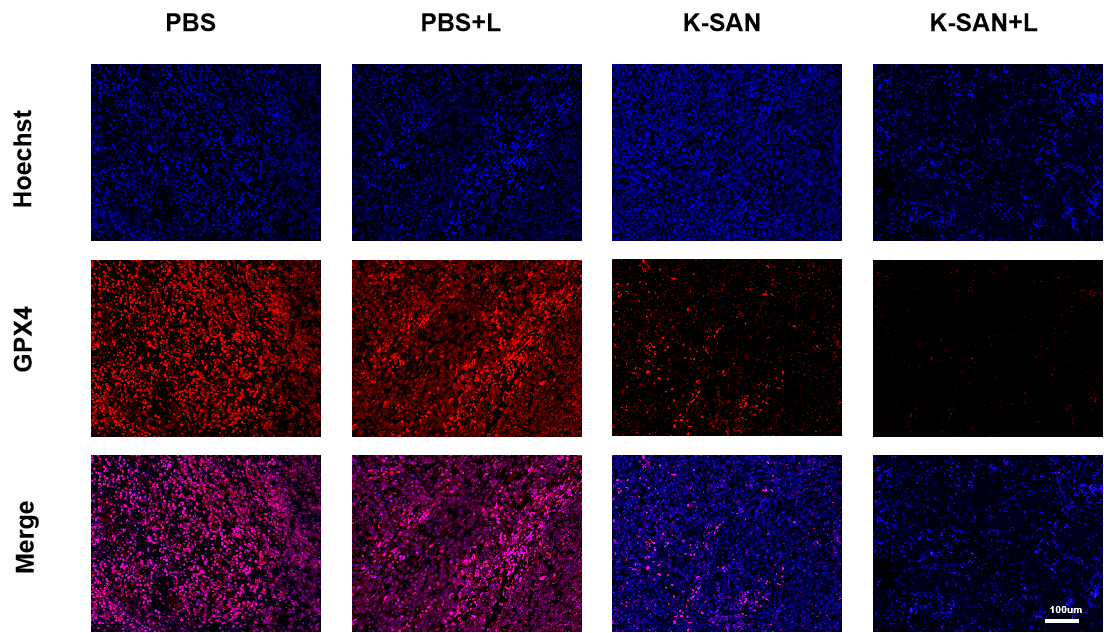


**Figure S39.** Immunofluorescence analysis of GPX4 protein expression in tumor treated with different formulation.


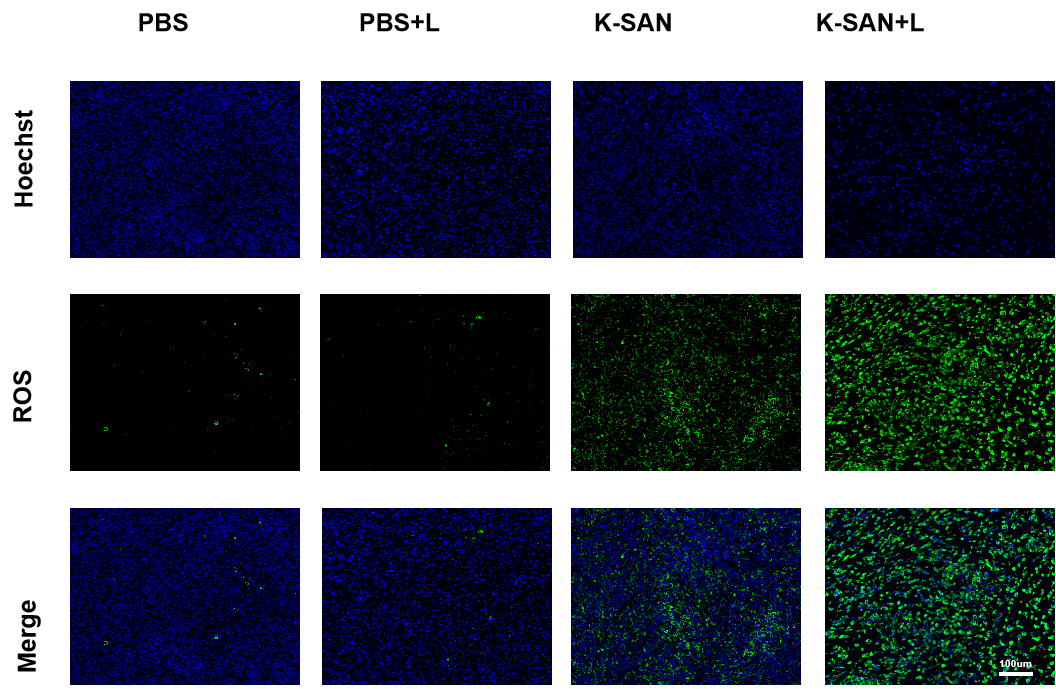


**Figure S40.** Immunofluorescence analysis of ROS expression in tumor treated with different formulation.
